# PLEKHH2 binds β-arrestin1 through its FERM domain, activates FAK/PI3K/AKT phosphorylation, and promotes the malignant phenotype of non-small cell lung cancer

**DOI:** 10.1038/s41419-022-05307-5

**Published:** 2022-10-08

**Authors:** Rui Wang, Si Wang, Zhen Li, Yuan Luo, Yue Zhao, Qiang Han, Xue-Zhu Rong, Yao-Xing Guo, Yang Liu

**Affiliations:** 1 Department of Pathology, College of Basic Medical Sciences and the First Hospital of China Medical University, Shenyang, 110122 P. R. China; 2 Department of Medical Microbiology and Human Parasitology, College of Basic Medical Sciences, China Medical University, Shenyang, 110122 P. R. China

**Keywords:** Non-small-cell lung cancer, Cell invasion, Growth factor signalling

## Abstract

PLEKHH2 is an important FERM domain containing-protein. However, the role of PLEKHH2 in human solid tumors has not been reported yet. We report that PLEKHH2 showed enhanced cytoplasmic expression in non-small cell lung cancer (NSCLC). Its overexpression was positively correlated with high TNM stage, low differentiation, lymphatic node metastasis, and poor prognosis. In A549 and H1299 cells, high expression of PLEKHH2 significantly promoted cell proliferation, migration, invasion, and increased the expression of proliferation- and invasion-related proteins. It also enhanced the phosphorylation of FAK and promoted the activity of the PI3K/AKT pathway. Immunofluorescence and co-immunoprecipitation analyses were performed to elucidate the molecular mechanism underlying PLEKHH2-mediated regulation of proliferation and invasion in lung cancer cells. Upon transfection of full length PLEKHH2 or its FERM domain, we observed enhanced binding of PLEKHH2 to β-arrestin1, whereas FAK- β-arrestin1 binding was diminished and this led to an increase in FAK phosphorylation. PLEKHH2-mutant plasmids without the FERM domain could not effectively promote its binding to β-arrestin1, activation of FAK phosphorylation, PI3K/AKT activation, or the malignant phenotype. Our findings suggested that PLEKHH2 is an important oncogene in NSCLC. PLEKHH2 binding to β-arrestin1 through the FERM domain competitively inhibits β-arrestin1 binding to FAK, which causes the dissociation of FAK from the FAK-β-arrestin1 complex. Furthermore, the dissociation of FAK promotes its autophosphorylation, activates the PI3K/AKT signaling pathway, and subsequently promotes lung cancer cell proliferation, migration, and invasion. These results provide evidence for the potential use of PLEKHH2 inhibition as an anticancer therapy.

## Introduction

Recurrence and metastasis are the main causes of death in lung cancer patients. Therefore, elucidating the molecular mechanisms underlying lung cancer cell proliferation and invasion could provide novel insights for the prevention and treatment of lung cancer recurrence and metastasis. Pleckstrin homology domain-containing family H member 2 (PLEKHH2) is an important member of the pleckstrin homology-like domain family. Aberrant expression of PLEKHH2 has been related to the occurrence and development of some diseases. Transcriptome analysis of fibroblasts from patients with schizophrenia has revealed differential expression of schizophrenia-related genes, including *PLEKHH2* [1]. Whole exome sequencing in thrombophilic pedigrees has identified PLEKHH2 as a genetic risk factor for venous thromboembolism [2]. However, the expression pattern and mechanism of action of PLEKHH2 in human malignant cancers has not yet been reported. A recent case report showed that PLEKHH2-ALK fusion is a novel oncogenic driver in non-small cell lung cancer (NSCLC) [3]. Hence, it would be interesting to explore the role and possible molecular mechanisms of PLEKHH2 in regulating the occurrence and development of lung cancer.

A unique domain organization is seen in PLEKHH2, consisting of two adjacent PH domains, a MyTH domain, and a FERM domain at the C-terminus. The N-terminal, comprising of the coiled-coil region, is sufficient for self-association of PLEKHH2 into a dimer. The PH and FERM domains cooperate to ensure proper localization. Recent reports have revealed that a functional and structural supramodule is formed by the interaction of the MyTH and FERM domains. A FERM-independent function for the MyTH domain of PLEKHH2 remains to be determined [4–6].

Because the C-terminus of PLEKHH2 contains the FERM domain (4.1-ezrin-radixin-moesin), we speculated that PLEKHH2 might be closely related to the PI3K/AKT or Ras/ERK signaling pathways. Many proteins containing the FERM domain interact with other structural domains and regulate the activity of the PI3K/AKT or Ras/ERK signaling pathways, thereby regulating cell proliferation, migration, and invasion [7–9]. In particular, many studies have confirmed that, in NSCLC, the abnormal activation of the PI3K/AKT and Ras/ERK signaling pathways plays a critical role in promoting the proliferation and invasion of lung cancer cells, as well as resistance to chemoradiotherapy and targeted therapy [10, 11]. The role of PLEKHH2 in regulating the PI3K/AKT or Ras/ERK signaling pathways in lung cancers has not been fully established yet.

By analyzing the SMART database, we found that proteins containing the FERM domain are mostly involved in focal adhesion assembly. Focal adhesion kinase (FAK) is recruited as an important participant in focal adhesion assembly and mediates PI3K/AKT and MAPK activation [12–14]. β-arrestins, including β-arrestin1 and β-arrestin2, were initially recognized for their roles in the desensitization and endocytosis of G protein-coupled receptors (GPCRs) [15]. Recently, it was reported that β-arrestins operate as on/off control switches for FAK activity and inhibit the autophosphorylation of FAK [16]. Whether β-arrestins and FAK are involved in PLEKHH2-mediated regulation of the PI3K/AKT or Ras/ERK signaling pathways in lung cancer is an open question.

In summary, this study aimed to explore the expression pattern of PLEKHH2 in NSCLC and its possible regulatory mechanism, providing new insights into the molecular mechanisms of lung cancer recurrence and metastasis.

## Results

### The overexpression of PLEKHH2 correlated with a malignant phenotype in patients with NSCLC

**Fig. 1:**
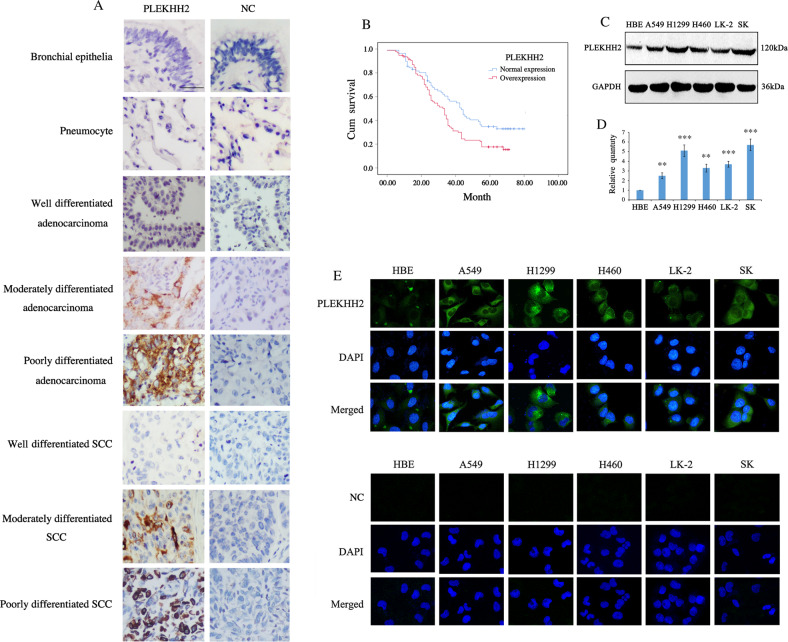
PLEKHH2 is upregulated in non-small cell lung cancer. **A** In normal bronchial and alveolar epithelia, negative PLEKHH2 staining was detected via immunohistochemical analysis. NSCLC samples showed cytoplasmic accumulation of PLEKHH2 protein, and it occurred more frequently in tumors with poorer differentiation (Rabbit IgG was used as a negative control; Scale bar, 50 μm). **B** Overexpression of PLEKHH2 correlates with poor patient survival. Among the 170 cases with follow-up information, Kaplan-Meier curves showed that patients with PLEKHH2 overexpression (94 cases) had a shorter survival time than those with normal PLEKHH2 expression (76 cases) (*P* = 0.014). **C** Western blot analysis of PLEKHH2 expression in lung cancer cells. Increased PLEKHH2 expression was detected in lung cancer cell lines compared to the immortalized bronchial epithelial cell line HBE. **D** PLEKHH2 protein levels were normalized to those of GAPDH. Statistical analysis showed increased PLEKHH2 expression in lung cancer cell lines compared to HBE cells. **E** Laser confocal microscopy images show that cytoplasmic PLEKHH2 expression was higher in lung cancer cells than in HBE cells. (PLEKHH2-CRISPR/CAS9 was used as negative control; Original magnification ×400). *P* < 0.05 indicates statistical significance, **P < 0.01, ***P < 0.001.

We performed immunohistochemical analysis of 197 NSCLC tissues to determine the PLEKHH2 expression patterns. PLEKHH2 was undetectable (-) or weakly positive in normal bronchial epithelia or pneumocytes. In 143 of 197 lung cancer specimens (72.59%), PLEKHH2 showed increased cytoplasmic expression (Fig. 1A). To determine the clinical significance of PLEKHH2 expression in NSCLC tissues, we analyzed the correlation between PLEKHH2 overexpression and clinicopathological parameters. As summarized in Supplementary Table 1, tumors with increased PLEKHH2 expression tended to display more malignant phenotypes such as lower differentiation (*P* = 0.001), greater clinical tumor size (*P* = 0.002), higher tumor node metastasis (TNM) stage (*P* = 0.013), and positive lymphatic metastasis (*P* = 0.002). In terms of survival, 170 of the 197 patients had complete follow-up information. Patients with PLEKHH2 overexpression had poorer overall survival than those with normal PLEKHH2 expression (*P* = 0.014, Fig. 1B, Supplementary Table 2). Western blot analysis (Fig. 1C, D), and immunofluorescence (Fig. 1E) were then performed in lung cancer cell lines, which showed increased PLEKHH2 expression compared to that in HBE cells. These results suggest that PLEKHH2 acts as a tumor promoter in NSCLC.

### PLEKHH2 promotes lung cancer cell growth and invasion

**Fig. 2:**
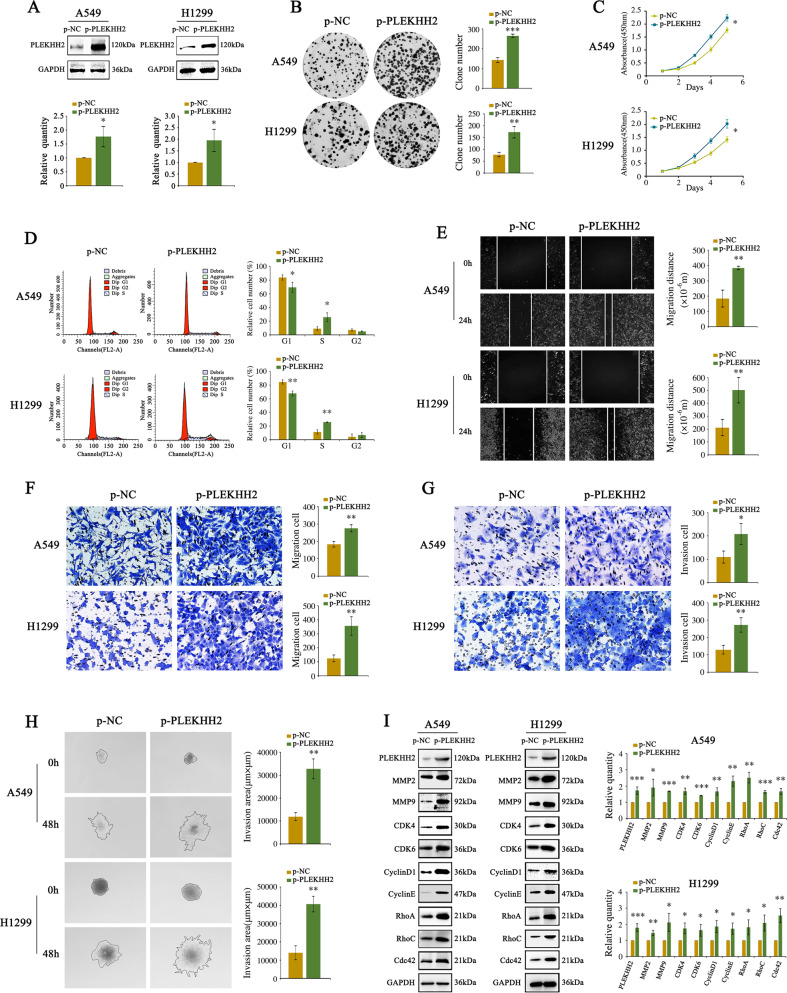
Enhanced PLEKHH2 expression promotes lung cancer cell proliferation, migration, and invasion. **A** Increased expression of PLEKHH2 was observed in A549 and H1299 cells transfected with the PLEKHH2-cDNA plasmid. Transfection efficiency was measured 48 h later via western blotting. **B** A significant increase in colony number was observed in cells with upregulated PLEKHH2 expression compared with the controls. **C** The CCK-8 assay showed a time-dependent increase in cell proliferation after PLEKHH2 plasmid transfection. **D** Flow cytometry results for the cell cycle analysis. Treatment with PLEKHH2 plasmids led to a significant decrease in the proportion of G-phase cells and a significant increase in S-phase cells compared with control cells. The scratch wound assay (**E**) and Transwell migration assay (**F**) revealed that cell migration was promoted in cells treated with the PLEKHH2 plasmid. **G** The Matrigel Transwell assay showed that cell invasion was significantly increased after PLEKHH2 transfection compared with the controls. **H** The results of the 3D invasion experiment showed that the invasion area of cells transfected with the PLEKHH2 plasmid was greater than that of the cells transfected with the control plasmid. **I** Cells transfected with PLEKHH2 plasmids showed increased expression of proliferation- and invasion-related proteins. *P* < 0.05 indicates statistical significance, *P < 0.05, **P < 0.01, ***P < 0.001.

**Fig. 3:**
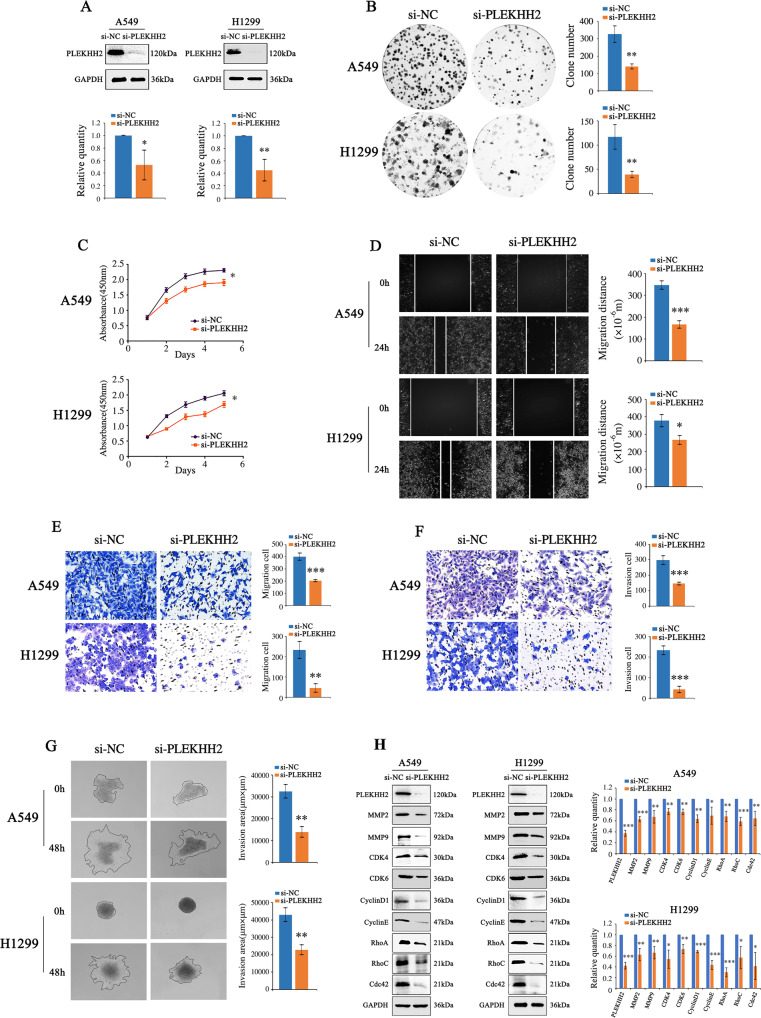
PLEKHH2 knockdown inhibits lung cancer cell growth and invasion. All three PLEKHH2-siRNAs down-regulated endogenous PLEKHH2 expression. **A** Cell lines transfected with PLEKHH2-siRNA plasmid showed decreased PLEKHH2 expression. Clone formation assay (**B**) and CCK-8 assay (**C**) showed that PLEKHH2-siRNA treatment led to a decrease in cell proliferation. The Scratch wound assay (**D**), Transwell migration assay (**E**), Matrigel Transwell assay (**F**), and 3D invasion experiment (**G**) showed that PLEKHH2 knockdown inhibited the biological functions of lung cancer cells, including migration and invasion. Western blotting showed that downregulation of PLEKHH2 decreased the expression of proliferation- and invasion-related proteins, including MMP2, MMP9, CDK4, CDK6, CyclinD1, CyclinE, RhoA, RhoC, and Cdc42 (**H**). The other two siRNAs showed similar changes, as shown in Supplementary Fig. 1 and Supplementary Fig. 2. *P* < 0.05 indicates statistical significance, *P < 0.05, **P < 0.01, ***P < 0.001.

We transfected a cDNA plasmid of PLEKHH2 into the adenocarcinoma cell lines A549 and H1299 (Fig. 2A). We observed that increased expression of PLEKHH2 increased the number of colonies and the size of lung cancer cells and accelerated cell growth (Fig. 2B, C). The upregulated expression of PLEKHH2 resulted in a remarkable decrease in the number of G1-phase cells and a significant increase in the number of S phase cells (Fig. 2D). Further experiments showed that increased PLEKHH2 expression increased the migration and invasion ability of lung cancer cells (Fig. 2E–H). Next, we examined the expression of proliferation-associated and invasion-associated proteins using western blotting. The results showed that increased PLEKHH2 levels resulted in increased expression of matrix metalloproteinases, cyclin, cyclin-dependent kinases, and Rho GTPase (Fig. 2I). After transfection with PLEKHH2-siRNAs in lung cell lines, we observed that PLEKHH2 silencing inhibited the proliferation, migration, and invasion of lung cancer cells and reduced the expression of proliferation-associated and invasion-associated proteins (Fig. 3, Supplementary Figs. 1 and 2). These results further confirmed that PLEKHH2 plays an important role in promoting the proliferation, migration, and invasion of lung cancer cells.

### PLEKHH2 promotes the proliferation and invasion of lung cancer cells by activating the PI3K/AKT signaling pathway

**Fig. 4:**
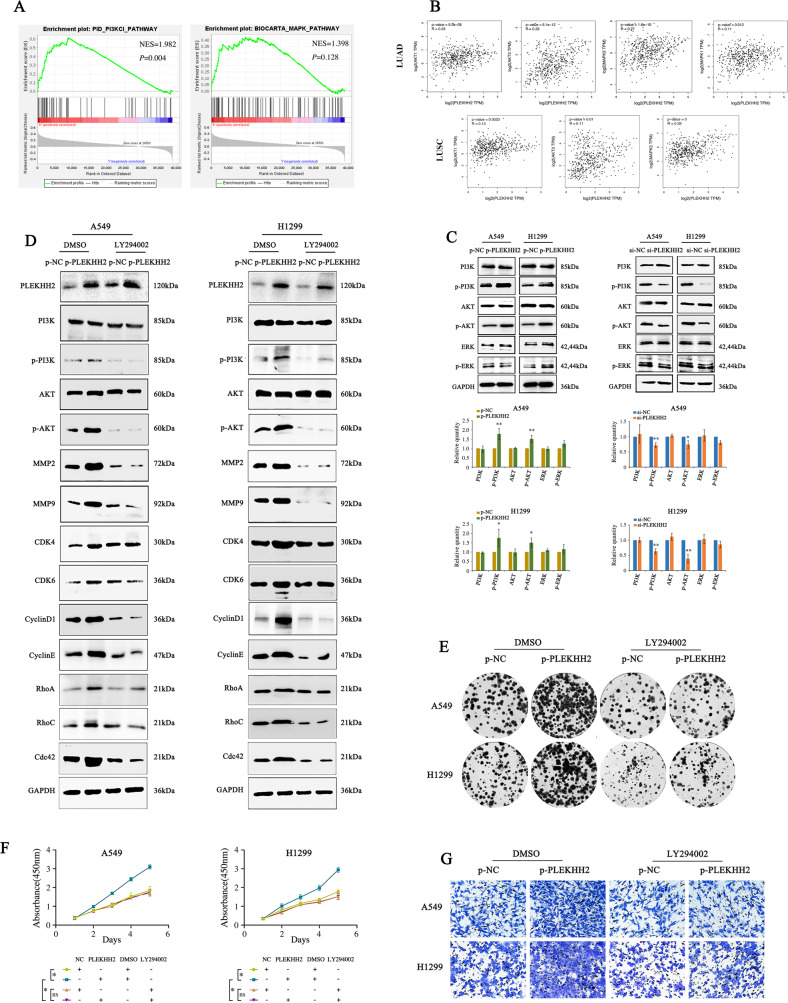
PLEKHH2 promotes the malignant phenotype of lung cancer cells by activating the PI3K/AKT signaling pathway. **A** GSEA analysis showed that, in lung adenocarcinoma, the expression of PLEKHH2 enriched in PI3K regulator activity in the TCGA cohort. **B** GEPIA analysis showed that PLEKHH2 was positively correlated with AKT and MAPK in lung adenocarcinoma (LUAD) and lung squamous cell carcinoma (LUSC). **C** The upregulation of PLEKHH2 significantly increased p-PI3K and p-AKT levels. The p-ERK level was not significantly upregulated, and the expression of total PI3K, AKT, and ERK did not change. In the cells with downregulated PLEKHH2 expression, p-PI3K and p-AKT levels were decreased, but p-ERK did not show a significant reduction. There was no significant change in total PI3K, AKT, and ERK proteins. **D** After inhibiting the PI3K signaling pathway via LY294002 treatment, an upregulation in PLEKHH2 expression did not significantly increase p-PI3K, p-AKT, and proliferation- and invasion-related proteins, as evidenced via western blotting. Statistical analysis in lung cancer cell lines is shown in Supplementary Fig. 4A and B. Results of the clone formation assay (**E**, Statistical analysis is shown in Supplementary Fig. 4C and D), CCK-8 assay (**F**), and Matrigel Transwell assay (**G**, Statistical analysis is shown in Supplementary Fig. 4E and F) showed that the upregulation PLEKHH2 expression had no significant effect on cell proliferation and invasion ability after treatment with LY294002. *P* < 0.05 indicates statistical significance, *P < 0.05, **P < 0.01.

Data analysis using the GSEA and GEPIA databases (Fig. 4A, B) showed that the high expression of PLEKHH2 is possibly correlated with the PI3K/AKT or MAPK signaling pathways. Here, we aimed to clarify whether PLEKHH2 has a regulatory effect on both the PI3K/AKT and Ras/ERK signaling pathways in lung cancer. As shown in Fig. 4C, PI3K/AKT was phosphorylated in lung cancer cells transfected with PLEKHH2 cDNA, whereas ERK phosphorylation did not show a significant increase. Decreased PLEKHH2 inhibited PI3K/AKT phosphorylation, whereas ERK phosphorylation did not show a clear decrease. Therefore, PLEKHH2 showed a significant regulatory effect on the activity of the PI3K/AKT signaling pathway but not on the activity of the ERK signaling pathway.

To further explore the role of the PI3K/AKT signaling pathway in PLEKHH2- mediated regulation of the proliferation and invasion of lung cancer cells, we used a PI3K/AKT pathway inhibitor (LY294002) to inhibit the activity of the PI3K/AKT signaling pathway and upregulate the expression of PLEKHH2. Next-generation sequencing of mRNA (RNA-seq) results showed that PLEKHH2 depletion or inhibitor treatment did not affect genes expression of PI3K, AKT, MMP2/9, CDK4/6, CyclinD1/E, and Rho GTPase (Supplementary Fig. 3). Western blot results showed that after inhibiting the PI3K/AKT signaling pathway, the enhanced expression of PLEKHH2 did not significantly promote the expression of proliferation- and invasion-related proteins (Fig. 4D, Supplementary Fig. 4A and B) and the proliferation and invasion abilities of lung cancer cells (Fig. 4E–G, Supplementary Fig. 4C–F). These results suggest that activation of the PI3K/AKT signaling pathway plays an important role in the PLEKHH2-mediated promotion of the malignant behavior of lung cancer cell lines.

### PLEKHH2 activates the PI3K/AKT signaling pathway by promoting FAK phosphorylation in lung cancer cells

**Fig. 5:**
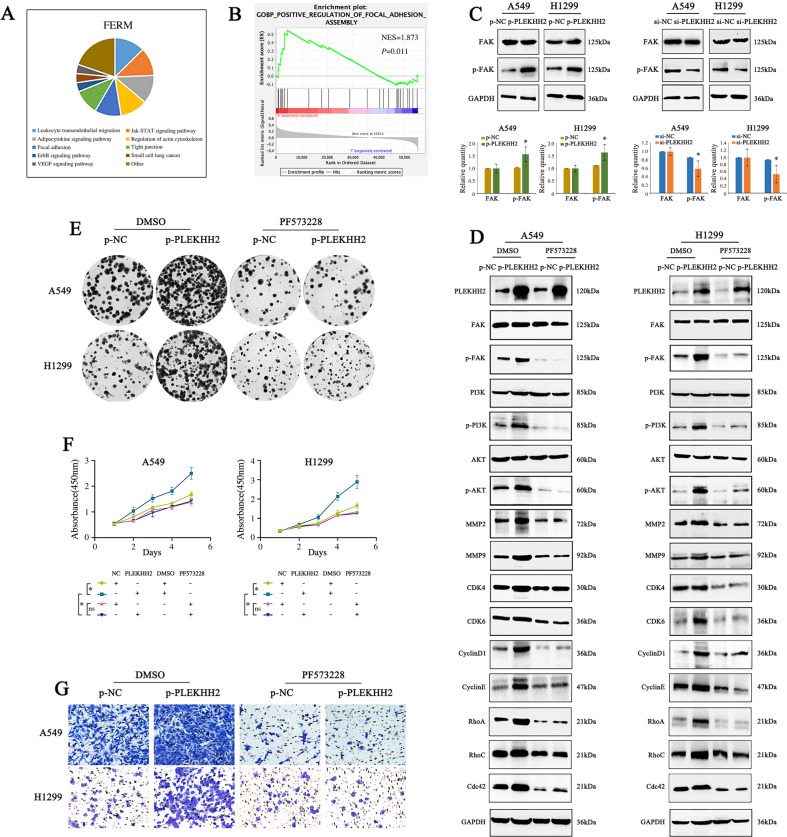
PLEKHH2 activates the PI3K/AKT signaling pathway by promoting FAK phosphorylation. **A** The SMART database was used to analyze the cellular functions of proteins containing the FERM domain. **B** The GSEA database predicted that PLEKHH2 was enriched in focal adhesion assemblies. **C** Overexpression of PLEKHH2 promotes FAK phosphorylation, which plays an important role in the focal adhesion assembly process. PLEKHH2 knockdown decreased FAK phosphorylation, but the total protein level of FAK did not change significantly. **D** After inhibiting the activity of the FAK via PF573228 treatment in A549 and H1299 cells, an increase in PLEKHH2 expression did not increase p-PI3K, p-AKT, or other proliferation-, migration-, and invasion-related proteins. Statistical analysis is shown in Supplementary Fig. 5A and 5B. Results of the clone formation assay (**E**, Statistical analysis is shown in Supplementary Fig. 5C and D), CCK-8 assay (**F**), and Matrigel Transwell assay (**G**, Statistical analysis is shown in Supplementary Fig. 5E and F) showed that, following the inhibition of FAK phosphorylation, the upregulation of PLEKHH2 did not significantly promote cell proliferation and invasion ability. *P* < 0.05 indicates statistical significance, *P < 0.05.

We further explored the molecular mechanism underlying PLEKHH2-mediated activation of the PI3K/AKT signaling pathway. After analyzing the SMART database (Fig. 5A), GSEA enrichment analysis (Fig. 5B), and the relevant literature, we investigated the possible relationship between PLEKHH2, FAK, and the PI3K/AKT signaling pathway. Western blot analysis revealed that the phosphorylation level of FAK increased after upregulating PLEKHH2. In contrast, decreased PLEKHH2 reduced the phosphorylated FAK (Fig. 5C). Next, we inhibited FAK phosphorylation using the FAK inhibitor PF573228. We found that upregulation of PLEKHH2 did not significantly promote the activity of the PI3K/AKT pathway or the proliferation and invasion of lung cancer cell lines (Fig. 5D–G, Supplementary Fig. 5). These results suggest that high expression of PLEKHH2 activates the PI3K/AKT signaling pathway by increasing FAK phosphorylation and promoting the proliferation, migration, and invasion of lung cancer cells.

### PLEKHH2 binds β-arrestins through its FERM domain, competitively inhibits the interaction between β-arrestins and FAK, promotes FAK phosphorylation

**Fig. 6:**
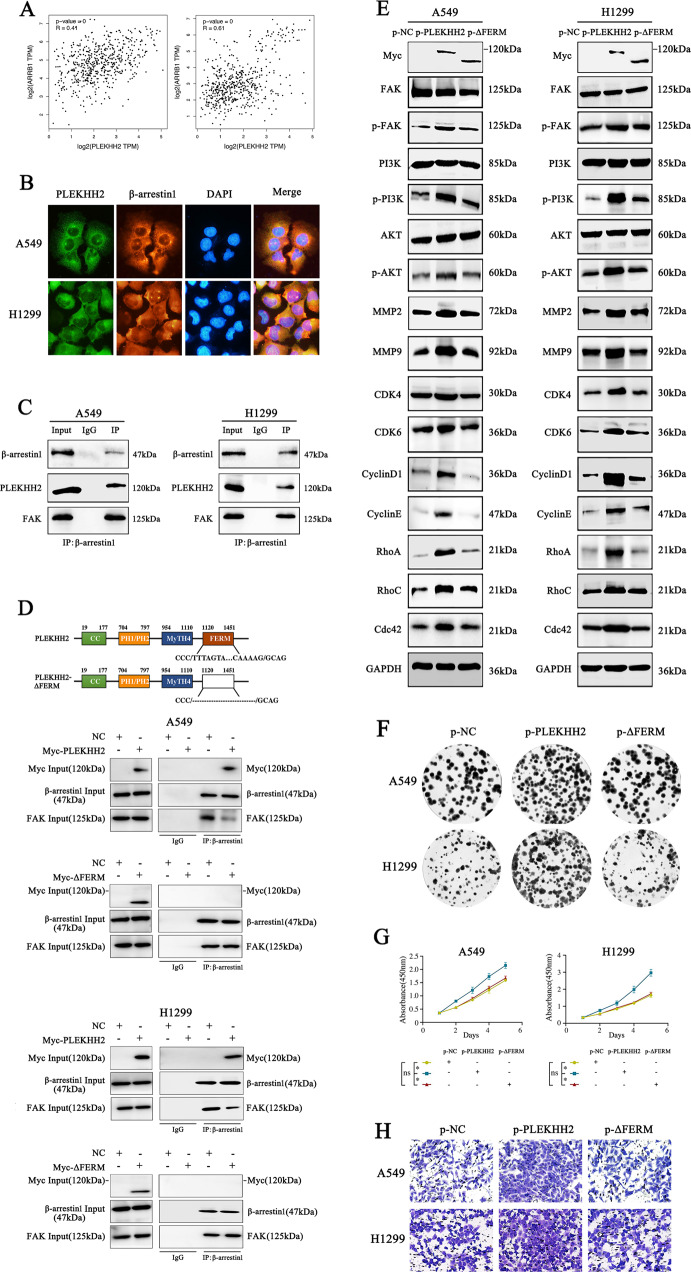
PLEKHH2 binds to β-arrestin1 through its FERM domain and promotes FAK phosphorylation, PI3K/AKT pathway activity, and cell malignant phenotype. **A** The GEPIA database showed that PLEKHH2 was significantly positively correlated with ARRB1 (β-arrestin1) level. **B** Endogenous β-arrestin1 and PLEKHH2 are co-localized in the cytoplasm. **C** Endogenous β-arrestin1 interact with both PLEKHH2 and FAK in A549 and H1299 cells. **D** In lung cancer cells, transfection with the full-length PLEKHH2 decreased the interaction of β-arrestin1 and FAK compared with that of the empty vector. Transfection with a PLEKHH2 mutant with a FERM domain deletion has no effect on decreasing β-arrestin1 binding to FAK. **E** Western blotting showed that PLEKHH2-ΔFERM did not significantly enhance p-FAK, p-PI3K, p-AKT, and proliferation- and invasion-related proteins compared to transfection with full-length PLEKHH2. At the same time, transfection with PLEKHH2-ΔFERM had did not significantly promote cell proliferation, migration, and invasion, as evidenced by the results of the clone formation assay (**F**), CCK-8 assay (**G**), and Matrigel Transwell assay (**H**). *P* < 0.05 indicates statistical significance, *P < 0.05. Statistical analysis is shown in Supplementary Fig. 6 and Supplementary Fig. 7A-B.

We then sought to determine how PLEKHH2 regulates FAK phosphorylation. The molecular structure of FAK also contains a FERM domain in its N-terminal fragment, which is required for FAK/β-arrestin interaction and FAK inhibition [16]. Since PLEKHH2 also has an FERM domain, we wondered if PLEKHH2 is associated with β-arrestins. We observed a positive correlation between PLEKHH2 and β-arrestin1 using the GEPIA database (Fig. 6A). Using immunofluorescence analysis (Fig. 6B), or co-IP (Fig. 6C), we found that endogenous PLEKHH2 and β-arrestin1 were located in the cytoplasm and interacted with each other. In addition, we confirmed the interaction between endogenous β-arrestin1 and FAK in lung cancer cells using co-IP. Therefore, we speculated that PLEKHH2 interacts with β-arrestin1 through its FERM domain and competitively inhibits the binding of β-arrestin1 to the FERM domain of FAK, reducing the inhibition of FAK autophosphorylation. To further verify our hypothesis, we transfected a PLEKHH2 mutant plasmid lacking the FERM domain into lung cancer cell lines.

Immunoprecipitation showed that the upregulation of PLEKHH2 decreased the binding of β-arrestin1 to FAK. In lung cancer cell lines transfected with the PLEKHH2 mutant plasmid, there was no association between β-arrestin1 and PLEKHH2, whereas the interaction of β-arrestin1 with FAK showed no significant variations (Fig. 6D). Furthermore, we found that compared with the full-length PLEKHH2 plasmid, the PLEKHH2 mutant plasmid had no effect on increasing FAK phosphorylation, PI3K/AKT pathway activity, or cell malignant phenotype (Fig. 6E–H, Supplementary Fig. 6).

**Fig. 7:**
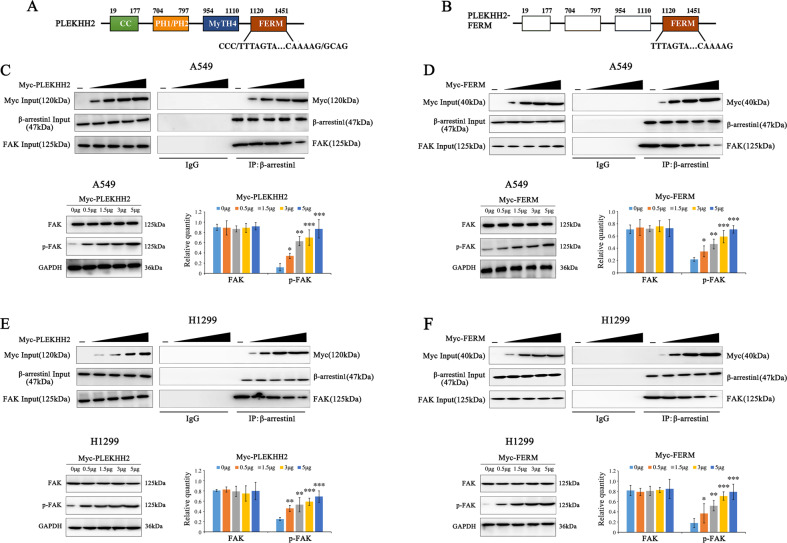
PLEKHH2 binds β-arrestin1 through its FERM domain, competitively inhibits β-arrestin1 interaction with FAK, and promotes FAK phosphorylation. **A, B** Schematic diagram of PLEKHH2 and PLEKHH2-FERM splicing mutants. Compared to the full-length PLEKHH2, PLEKHH2-FERM only has the isolated FERM domain. **C** PLEKHH2 overexpression attenuates the binding between β-arrestin1 and FAK. In the A549 cell line, the PLEKHH2 plasmid (0 μg, 0.5 μg, 1.5 μg, 3 μg, and 5 μg) was transfected in a dose-dependent manner. β-arrestin1 was used for immunoprecipitation. The binding of β-arrestin1 to PLEKHH2 gradually increased, while the association of β-arrestin1 and FAK was gradually decreased (Statistical analysis is shown in Supplementary Fig. 7E). Moreover, PLEKHH2 overexpression increased FAK phosphorylation gradually. **D** FERM domain (0 μg, 0.5 μg, 1.5 μg, 3 μg, and 5 μg) was transfected into A549 cells. It exhibited gradually increase in binding with β-arrestin1 and gradually decrease in binding with β-arrestin1 and FAK (Statistical analysis is shown in Supplementary Fig. 7F). It also promoted p-FAK level in a dose gradient manner. Both PLEKHH2 and its FERM had no effect on FAK expression. Similar results are seen in H1299 cell line (**E, F**, Supplementary Fig. 7G and H). Increased p-FAK was compared to the cells with no transfection. *P* < 0.05 indicates statistical significance, *P < 0.05, **P < 0.01, ***P < 0.001.

To further test the competitive binding of β-arrestin1 with PLEKHH2 and FAK, we transfected a PLEKHH2 mutant plasmid containing only the FERM domain into lung cancer cell lines. Immunoprecipitation revealed that in response to PLEKHH2 or its FERM domain overexpression in a dose-dependent manner, the binding of β-arrestin1 and PLEKHH2 gradually increased, while the binding of β-arrestin1 to FAK gradually decreased. FAK activity was also increased in a dose-dependent manner (Fig. 7, Supplementary Fig. 7).

These results confirm that PLEKHH2 competes with β-arrestin1 through its FERM domain and reduces the association of β-arrestin1 with FAK. This reduced interaction, in turn, reduces the inhibition of FAK phosphorylation by β-arrestin1, promoting the phosphorylation of FAK.

## Discussion

In this study, we explored the role of PLEKHH2 and the possible mechanisms by which it promotes the proliferation and invasion of lung cancer cells, mediated by its FERM domain.

Most proteins containing a FERM domain have been reported to play important roles in regulating the activity of the PI3K/AKT and Ras/ERK signaling pathways. First, we aimed to clarify whether PLEKHH2 expression plays an important role in regulating both the PI3K/AKT and Ras/ERK pathways and promoting the malignant phenotype of lung cancer. Our results show that PLEKHH2 significantly activated PI3K/AKT phosphorylation, whereas ERK phosphorylation was not altered. Although GEPIA database analyses revealed a correlation between PLEKHH2 expression and the MAPK signaling pathway, we have not confirmed the relevance of PLEKHH2 expression in ERK/MAPK activation. The Ras/ERK pathway is known to be the most important signaling cascade among all MAPK signal transduction pathways, JNK, p38 MAPK, and ERK5 being the other relevant MAPK cascades. All these factors play important roles in cancer development and progression [17, 18]. There is a possible relationship between PLEKHH2 expression and other MAPK signaling pathways; however, this relationship needs to be further tested.

In the current study, we addressed PLEKHH2 expression and the PI3K/AKT pathway in greater detail. After treatment with the PI3K/AKT pathway inhibitor LY294002, the upregulated PLEKHH2 did not significantly promote cell proliferation, migration, or invasion. Therefore, we established that PLEKHH2 promotes the malignant phenotype of lung cancer cells by activating the PI3K/AKT signaling pathway. We further explored the molecular mechanism by which PLEKHH2 regulates the PI3K/AKT signaling pathway. By analyzing the SMART database, we found that proteins containing the FERM domain were mostly involved in focal adhesion assembly. FAK, a highly conserved non-receptor tyrosine kinase, is a key signaling mediator upstream of PI3K [19, 20]. Autophosphorylation of FAK at Tyr397 allows direct interaction with the SH2 domain of p85, the regulatory subunit of PI3K. Activated PI3K triggers downstream signaling pathways that control cell adhesion, polarity, motility, proliferation, and survival [21–25]. Furthermore, GSEA enrichment analysis implied that the expression of PLEKHH2 was correlated with focal adhesion assemblies. Therefore, we determined whether PLEKHH2, a protein containing the FERM domain, activates FAK and, in turn, activates the PI3K/AKT signaling pathway. The results showed that PLEKHH2 upregulation promoted FAK phosphorylation in lung cancer cell lines. After inhibiting FAK phosphorylation, PLEKHH2 upregulation did not significantly increase PI3K/AKT activity or cell proliferation and invasion. Therefore, we confirmed that PLEKHH2 promotes the activity of the PI3K/AKT signaling pathway by promoting FAK phosphorylation, which then confers a malignant phenotype in lung cancer cells.

Finally, we explored the molecular mechanism by which PLEKHH2 regulated FAK phosphorylation. The main effect of FAK activity is the autophosphorylation of Tyr397, which is located between the FERM and central kinase domains [24]. In the cytoplasm of quiescent cells, intramolecular interactions between FERM and kinase domains prevent accessibility to Tyr397, blocking the catalytic site and maintaining FAK in an inactive state [26–29]. β-arrestins are known to act as multifunctional adaptor proteins that bind many non-receptor protein partners to control multiple signaling pathways. β-arrestins stabilize the interaction between the FERM and kinase domains, preventing FAK “opening,” and the FAK FERM domain is sufficient for its association with β-arrestins. Both β-arrestin isoforms interact with FAK and appear to contribute to FAK inhibition [16]. Since PLEKHH2 is also a protein with a FERM domain, could it interact with β-arrestins?

In the present study, we examined the interaction between PLEKHH2 and β-arrestin1. After confirming the endogenous interaction of β-arrestin1 with PLEKHH2 and FAK in lung cancer cells, we transfected the full-length PLEKHH2, PLEKHH2 lacking the FERM domain, and isolated the FERM domain of PLEKHH2. Then, using immunocoprecipitation, we examined the change in the association of β-arrestin1 with PLEKHH2 and FAK. Our results show that PLEKHH2 competitively binds to β-arrestin1 via its FERM domain, inhibits the interaction between β-arrestin1 and FAK, and promotes FAK phosphorylation. The results further showed that PLEKHH2 without the FERM domain has a weakened ability to activate the PI3K/AKT pathway and, in turn, the ability to promote lung cancer cell proliferation and invasion.

The apparent propensity of association with FAK has been reported to be similar for β-arrestin1 and β-arrestin2 [16]. It was also reported that β-arrestin1-STAM1 (signal-transducing adaptor molecule 1) complex promotes FAK autophosphorylation, while β-arrestin2 does not interact with STAM1. It is possible that β-arrestin1 regulates cell migration via STAM1 and FAK, whereas β-arrestin2 operates via a distinct mechanism [30]. In our study, β-arrestin2 was not studied. Therefore, whether β-arrestin2 has an effect similar to that of β-arrestin1 in PLEKHH-dependent regulation of the FAK phosphorylation remains to be seen. Further investigations are required to address this issue.

Other proteins have also been reported to interact with FERM domains. ERM proteins (ezrin/radixin/moesin) bind to the cytoplasmic domain of CD44 through their FERM domain. Phosphorylated ERM plays a vital role in the regulation of cell proliferation, migration, and invasion [31]. The FERM domain of Janus kinase (JAK) mediates its binding to the cytokine receptor gp130 [32]. JAK then becomes activated, phosphorylating tyrosine residues in the cytoplasmic tail of receptors, leading to the recruitment and activation of signal transducer and activator of transcription (STAT) and other signaling molecules [33]. Whether PLEKHH2 also binds to these proteins through its FERM domain and plays other regulatory roles in lung cancer remains unknown and could be a subject of future research.

**Fig. 8:**
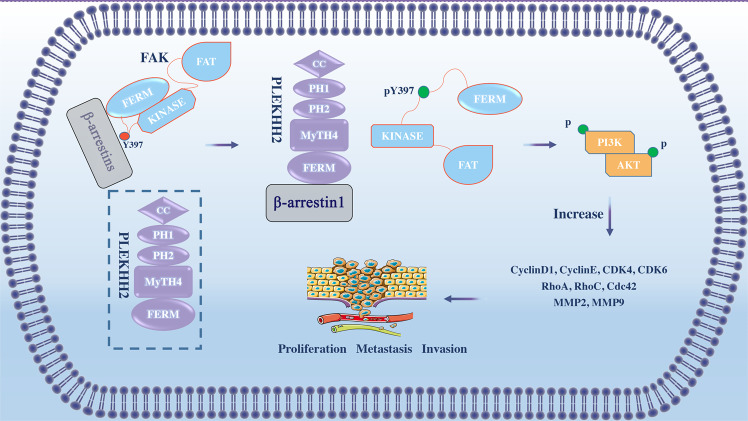
A model depicting the proposed mechanism of PLEKHH2. Schematic model for the regulation of FAK/PI3K/AKT phosphorylation activity by PLEKHH2 in nonsmall cell lung cancer using Figdraw (www.figdraw.com).

In the present study, we demonstrated that PLEKHH2 plays a vital role in promoting the malignant phenotype of lung cancer. PLEKHH2, through its FERM domain, binds to β-arrestin1 and competitively inhibits the interaction between β-arrestin1 and FAK, which causes FAK dissociation from the FAK-β-arrestin1 complex. This dissociation reverses the β-arrestin1-mediated inhibition of FAK autophosphorylation, promoting the autophosphorylation of FAK, activating the PI3K/AKT signaling pathway, and promoting the proliferation, migration, and invasion of non-small cell lung cancer cells (Fig. 8).

Simultaneously, through analysis of the Gene Expression Omnibus (GEO) database, we also found that the expression of PLEKHH2 in EGFR-TKI-resistant cell lines was significantly higher than that in EGFR-TKI-sensitive cell lines (Supplementary Fig. 8, *P* < 0.001). Although the mechanisms of EGFR-TKI resistance are complex and incompletely understood, abnormal activation of PI3K/AKT is the explicit mechanism leading to EGFR-TKI resistance in lung cancer cells [34–36]. Increased PLEKHH2 binding to β-arrestin1 through its FERM domain activates FAK/PI3K/AKT phosphorylation. Therefore, PLEKHH2, acting as a regulator of the PI3K/AKT signaling pathway, may play an important role in EGFR-TKI resistance in patients with NSCLC. However, whether the potential mechanisms related to PLEKHH2 in this study also play an important role in EGFR-TKI resistance still needs to be verified in future studies.

## Materials and methods

### Specimen collection

This study was approved by the ethics committee of the China Medical University and was performed according to the requirements of the Declaration of Helsinki. All patients with lung cancer who participated in this study were aware of the study and signed an informed consent form. Primary tumor specimens were obtained from 197 patients (91 male and 106 female) who underwent complete resection at the First Affiliated Hospital of China Medical University. According to the 2015 World Health Organization (WHO) histopathological diagnostic criteria for lung cancer, the samples obtained were identified as adenocarcinoma in 117 cases and squamous cell carcinoma (SCC) in 80 cases. According to the American Joint Committee on Cancer tumor-node-metastasis (TNM) staging criteria published by the International Association for the Study of Lung Cancer in 2017 (8^th^ edition), 126 cases were identified as stages I–II and 71 cases as stage III. None of the patients was treated with radiotherapy, chemotherapy, or tyrosine kinase inhibitors (TKIs) before definitive pathological diagnoses were determined.

### Immunohistochemistry (IHC)

Briefly, tissue sections were incubated with rabbit polyclonal anti-PLEKHH2 antibody (1:150; Proteintech Group, Inc.; Wuhan Sanying, China). Rabbit immunoglobulin (1:200; Beyotime Biotechnology, Shanghai, China) was used as a negative control and biotinylated goat anti-rabbit immunoglobulin G (IgG; Beyotime Biotechnology) was used as the secondary antibody. After washing, the sections were incubated with streptavidin-biotin-conjugated horseradish peroxidase and developed with 3,3′-diaminobenzidine tetrahydrochloride (Beyotime Biotechnology). Positive PLEKHH2 expression was determined based on the intensity of cytoplasmic staining of tumor cells and the proportion of PLEKHH2-positive cells in the tissue sections. Two independent investigators evaluated the staining in a blinded manner by randomly selecting five fields of view per slide at 400× magnification, with each field of view containing at least 100 cells. The staining intensity was scored as 0 (no signal), 1 (weak), or 2 (strong). The percentage of stained cells was scored as 1 (1–25%), 2 (26–50%), 3 (51–75%), or 4 (76–100%). The scores for staining intensity and percentage of cells stained in each tumor sample were multiplied to obtain a final score of 0–8. Tumors were classified as negative (score 0), low-expression (score 1-3), or high-expression (score ≥ 4). Tumor samples with a cytoplasmic staining score of 2+ were considered positive (overexpression).

### Cell culture

All the cell lines were purchased from the Cell Bank of the Chinese Academy of Sciences (Shanghai, China). Human bronchial epithelial cells (HBEs), human adenocarcinoma cell lines (A549, LTEP-a-2, H1299), and SCC cell lines (LK-2, SK-MES-1) were cultured in Dulbecco’s modified Eagle’s medium or Roswell Park Memorial Institute 1640 medium (both from Invitrogen, Carlsbad, CA, USA) supplemented with 10% fetal calf serum (Invitrogen), 100 IU/mL penicillin (Sigma-Aldrich, Inc., St. Louis, MO, USA), and 100 μg/mL streptomycin (Sigma) at 37 °C in 5% CO_2_. All cell lines were authenticated using short tandem repeat DNA profiling and tested for mycoplasma contamination.

### Western blot analysis

Total protein concentration was determined using a bicinchoninic acid (BCA) kit (Pierce Biotechnology Inc., Rockford, IL, USA). Equal amounts of cell lysate were separated by sodium dodecyl sulfate-polyacrylamide gel electrophoresis, and proteins were detected by western blotting. Primary antibodies against PLEKHH2 (14204-1-AP, 1:1000) and β-arrestin1 (15361-1-AP, 1:1000) were purchased from Proteintech. Antibodies against RhoA (sc-418, 1:300), RhoC (sc-393090, 1:300), Cdc42 (sc-8401, 1:300), CyclinD1 (sc-8396, 1:200), cyclin E (sc-377100, 1:200), MMP2 (sc-13595, 1:300), MMP9 (sc-393859, 1:300), CDK4 (sc-23896, 1:300), CDK6 (sc-7961, 1:300), ERK1/2 (sc-514302, 1:300), and phospho-ERK1/2 (sc-81492,1:300) were purchased from Santa Cruz Biotechnology (Santa Cruz, CA, USA). Antibodies against PI3K (4292, 1:500), phospho-PI3K (4228, 1:500), AKT (4691, 1:500), phospho-AKT (4060, 1:500), and GAPDH (5174, 1:10000) were purchased from Cell Signaling Technology (CST Inc., Danvers, MA, USA). Primary antibodies against FAK (ab40794, 1:1000) and phospho-FAK (Y397) (ab81298, 1:1000) were purchased from Abcam (Cambridge, MA, USA). Secondary antibodies were purchased from CST Inc. Protein expression was quantified using densitometry and ImageJ software, and protein bands were visualized using an ECL western blotting substrate (Pierce) and a BioImaging System (UVP Inc., Upland, CA, USA). GAPDH was used as an internal control for normalization.

### Cell transfection

PLEKHH2-siRNAs (three different sequences were used to downregulate endogenous PLEKHH2 expression), PLEKHH2-cDNA, PLEKHH2-ΔFERM-cDNA (deletion position 1120-1451), PLEKHH2-FERM-cDNA (only position 1120-1451), and negative controls were purchased from Miaoling Biological Technology Co. Ltd. (Wuhan, China). The cells were transfected with plasmids using the Attractene Transfection Reagent (Qiagen Inc., Hilden, Germany) according to the manufacturer’s instructions. Transfection efficiency was determined by western blotting.

PLEKHH2-specific CRISPR/CAS9 was purchased from Miaoling Biological Technology and transfected using Lipofectamine 3000 (Qiagen) according to the manufacturer’s instructions.

### Immunofluorescence staining

Cells were fixed, permeabilized, and incubated with primary antibodies against PLEKHH2, β-arrestin1, and fluorescein isothiocyanate-conjugated (FITC) or tetramethylrhodamine isothiocyanate-conjugated (TRITC) secondary antibodies (Beyotime Biotechnology). Nuclei were stained with 4’,6-diamidino-2-phenylindole, and cells were observed using a confocal microscope (Carl Zeiss, Olympus, Tokyo, Japan).

### Colony formation assay

Cells were plated in 6-cm cell culture dishes (500 cells per dish) and incubated for 14 days. The cells were washed with phosphate-buffered saline, fixed with 4% paraformaldehyde, and stained with Giemsa stain. Colonies containing >50 cells were counted under a microscope. Microscopic images were obtained, and the number of colonies was counted in each image.

### CCK-8 assay

A CCK-8 kit (GlpBio Technology, Montclair, CA, USA) was used to measure the cell proliferation as per the manufacturer’s instructions. A total of 1000 cells per 100 μL were cultured per well in a 96-well plate. Then, 10 μL of the CCK-8 reagent was added to each well. This assay was performed at 0, 24, 48, 72, 96, and 120 h. At each time point, the OD_450_ was determined using a microplate reader (Bio-Rad Laboratories, Hercules, CA, USA).

### Flow cytometry

After 48 h of culture, the cells harvested from each experimental group were resuspended in 50 μg/mL propidium iodide (Sigma) and incubated for 45 min at room temperature in the dark, prior to fluorescence-activated cell sorting analysis. The proportion of cells at each cell cycle phase was determined using a FACS Calibur Flow Cytometer and CellQuest 3.0 software (BD Biosciences, San Jose, CA, USA).

### Wound healing assay

Cells (1 × 10^5^/well) were plated in each well of a 6-well plate. After reaching confluence, cell monolayers were scratched in a straight line at the center of each well using a sterile pipette tip. PBS was added to wash the cells and remove cell debris. Photographs of the scratch wounds were recorded at 6, 12, and 24 h. Digital images were obtained using an inverted microscope (Olympus), and the scratch area was measured using the ImageJ software.

### Transwell migration and invasion assay

For the migration assay, 200 μL of cell suspension (containing 6 × 10^4^ cells) was added to the upper chamber and 600 μL of culture medium containing 20% serum was added to the lower chamber of each Transwell insert. The cells and the inserts were then incubated at 37 °C for 24 h. Cells in the inner layer of the chamber were removed using a cotton swab, fixed with methanol for 15 min, and stained with 0.1% crystal violet dye for 10 min. Finally, cells were counted at 100× magnification and photographed using a microscope (Olympus).

For the invasion experiment, cells were grown in serum-free medium on membranes (Corning, Acton, MA, USA) coated with Matrigel basement membrane matrix (BD Biosciences) in the upper chamber of each Transwell insert. The remaining steps were the same as those used for the migration experiments.

### 3D cell invasion assay

The cells were resuspended in fresh cell culture medium to obtain single-cell suspensions. The cell density was then adjusted to 2.5 × 10^4^ cells/mL. The lid of the culture dish was inverted and a cell suspension (20 μL) was gently added to the dish lid. Then, 6 mL of phosphate-buffered saline (PBS) was added to the dish, and the dish was covered with a lid. The dish was placed in a humidified incubator (37 °C, 5% CO_2_) to cultivate multicellular tumor spheroids. The dish was observed every three days, and the culture medium was replaced, if necessary. Next, 40 μL of a cell suspension containing tumor spheroids was added to a mixture of Matrigel and collagen, and 40 μL of the mixture was dropped into each well of a 24-well plate. After solidification, 1 mL of RPMI 1640 medium containing 10% fetal calf serum was pipetted into each well. Images were obtained using a microscope (Olympus) at 0, 24, and 48 h of incubation, and the total invasive area in vitro was analyzed using ImageJ software.

### Bioinformatics analysis

Signaling pathways potentially related to *PLEKHH2* were detected using gene set enrichment analysis (GSEA, version 4.1.0, http://software.broadinstitute.org/gsea/index.jsp). Data from “c5.go.v7.4. symbols.gmt) was used as a reference gene set. The threshold for enrichment significance was set at a NOM *p* value < 0.05. Gene expression levels were obtained and visualized using the GEPIA database (https://gepia.cancer-pku.cn/). The functions of proteins containing the FERM domain were then comprehensively analyzed using the SMART database (http://smart.embl-heidelberg.de/), which identifies and annotates genetically mobile domains and analyzes domain architecture.

### Phosphorylation inhibitor assay

For the phosphorylation inhibitor assay, cells were plated in 6-well tissue culture plates and allowed to attach overnight. PF-573228 (10 μM, SC1099, Beyotime) or LY294002 (40 μM, HY-10108, Med Chem Express) was dissolved in dimethyl sulfoxide (DMSO, Invitrogen). The vehicle DMSO was used in parallel experiments and the cells were harvested after 48 h.

RNA-seq was performed by Sangon Biotechnology Co., Ltd. (Shanghai, China) in the cells with PLEKHH2-siRNA and the cells treated with inhibitor LY294002.

### Co-immunoprecipitation (Co-IP)

Cell lysates were centrifuged at 14 000 g, and protein concentration was determined using a BCA kit (Pierce Biotechnology). Equal amounts of cell lysate (400 μg/group) were divided into the input, IgG, and IP groups. Then incubated with protein A + G agarose (20 μl, Beyotime Biotechnology) and rabbit IgG (1 μg, Beyotime Biotechnology) for 2 h at 4 °C to remove the nonspecific binding. After centrifugation at 1000 g for 5 min, the supernatant was collected and incubated overnight at 4 °C with 2 μg rabbit β-arrestin1 or a control rabbit IgG. 20 μl Protein A + G Agarose was added and incubated for additional 3 h at 4 °C. After centrifugation (1000 × *g*), the beads were collected and washed in cold PBS three times. The samples were subjected to western blotting using anti-Myc, β-arrestin1, PLEKHH2, and FAK antibodies. The experiment was repeated twice, and the input group was used as the internal control for normalization.

### Statistical analysis

SPSS (version 24.0; IBM Corp., Armonk, NY, USA) was used for all statistical analyses. Correlations between PLEKHH2 expression and clinicopathological parameters of the patients were assessed using the chi-square test. Survival curves were generated using the Kaplan–Meier method. Each cell line experiment was performed in triplicate. Western blot gray values were detected using Image Lab^TM^ (Bio-Rad Laboratories) and compared using the Student’s t-test. *P*-values less than 0.05 (two-sided) indicated significance. The columns represent mean values and bars SD.

## Supplementary information

Supplementary Table 1,

Supplementary Table 2,

Supplementary Figure Legends

Supplementary Figure 1,

Supplementary Figure 2,

Supplementary Figure 3,

Supplementary Figure 4,

Supplementary Figure 5,

Supplementary Figure 6,

Supplementary Figure 7,

Supplementary Figure 8,

Uncropped western blot-Fig1C

Uncropped western blot-Fig2A

Uncropped western blot-Fig2I

Uncropped western blot-Fig3A

Uncropped western blot-Fig3H

Uncropped western blot-Fig4C

Uncropped western blot-Fig4D

Uncropped western blot-Fig5C

Uncropped western blot-Fig5D

Uncropped western blot-Fig6C

Uncropped western blot-Fig6D

Uncropped western blot-Fig6E

Uncropped western blot-Fig7

Uncropped western blot-Supplementary Fig1G

Uncropped western blot-Supplementary Fig2G

aj-checklist

## Data Availability

The datasets supporting the conclusions of this article are included within the article and its additional files.
