# Supplementary material for: PLEKHH2 binds β-arrestin1 through its FERM domain, activates FAK/PI3K/AKT phosphorylation, and promotes the malignant phenotype of non-small cell lung cancer
Source: Cell Death Dis. 2022 Oct 8;13(10):858. doi: 10.1038/s41419-022-05307-5 (PMC9547923; doi:10.1038/s41419-022-05307-5)
Supplement: Supplementary file 1 — Supplementary Table 1, [file 41419_2022_5307_MOESM1_ESM.docx]

| **Clinicopathological**  **Feature** | **N (197)** | **Overexpression**  **(143)** | **ϰ2** | ***p*-value** |
| --- | --- | --- | --- | --- |
| **Age(years)** |  |  |  |  |
| ＜54 | 87 | 60 (68.97%) | 1.028 | 0.311 |
| ≥54 | 110 | 83 (75.45%) |  |  |
| **Gender** |  |  |  |  |
| Male | 91 | 70 (76.92%) | 1.597 | 0.206 |
| Female | 106 | 73 (68.87%) |  |  |
| **Histological Type** |  |  |  |  |
| squamous-cell carcinoma | 80 | 62 (77.50%) | 1.633 | 0.201 |
| adenocarcinoma | 117 | 81 (69.23%) |  |  |
| **Differentiation** |  |  |  |  |
| Well | 98 | 61 (62.24%) | 10.487 | 0.001 |
| Moderate & Poor | 99 | 82 (82.83%) |  |  |
| **Tumor status** |  |  |  |  |
| T1 | 119 | 77 (64.71%) | 9.386 | 0.002 |
| T2, T3, T4 | 78 | 66 (84.62%) |  |  |
| **TNM classification** |  |  |  |  |
| I+II | 126 | 84 (66.67%) | 6.162 | 0.013 |
| III | 71 | 59 (83.10%) |  |  |
| **Lymph node metastasis** |  |  |  |  |
| Positive | 82 | 69 (84.15%) | 9.430 | 0.002 |
| Negative | 115 | 74 (64.35%) |  |  |

**Supplementary Table 1, Overexpression of PLEKHH2 correlated with malignant phenotype in NSCLC patients**

TNM: tumor node metastasis
