# Supplementary material for: PLEKHH2 binds β-arrestin1 through its FERM domain, activates FAK/PI3K/AKT phosphorylation, and promotes the malignant phenotype of non-small cell lung cancer
Source: Cell Death Dis. 2022 Oct 8;13(10):858. doi: 10.1038/s41419-022-05307-5 (PMC9547923; doi:10.1038/s41419-022-05307-5)
Supplement: Supplementary file 2 — Supplementary Table 2, [file 41419_2022_5307_MOESM2_ESM.docx]

**Supplementary Table 2, Case processing summary for survival curves in NSCLC patients**

| **Group**  **PLEKHH2 expression** | **Total N** | **N of Events** | **Censored** | |
| --- | --- | --- | --- | --- |
|  |  |  | N | Percent |
| **Normal expression** | 76 | 47 | 29 | 38.2% |
| **Overexpression** | 94 | 74 | 20 | 21.3% |
| **Overall** | 170 | 121 | 49 | 28.8% |
