# Supplementary material for: PLEKHH2 binds β-arrestin1 through its FERM domain, activates FAK/PI3K/AKT phosphorylation, and promotes the malignant phenotype of non-small cell lung cancer
Source: Cell Death Dis. 2022 Oct 8;13(10):858. doi: 10.1038/s41419-022-05307-5 (PMC9547923; doi:10.1038/s41419-022-05307-5)
Supplement: Supplementary file 3 — Supplementary Figure Legends [file 41419_2022_5307_MOESM3_ESM.docx]

**Supplementary Figure Legends**

**Supplementary Figure 1. Cell lines transfected with the second PLEKHH2-siRNA inhibited lung cancer cell growth and invasion.**

Clone formation assay **(A)** and CCK-8 assay **(B)** showed that decreased PLEKHH2 inhibited the cell proliferation. The Scratch wound assay **(C)**, Transwell migration assay **(D)**, Matrigel Transwell assay **(E)**, and 3D invasion experiment **(F)** showed that PLEKHH2 knockdown inhibited the cell migration and invasion. **(G)** Western blotting showed that the second PLEKHH2-siRNA effectively down-regulated endogenous PLEKHH2 expression. PLEKHH2 downregulation decreased the expression of proliferation- and invasion-related proteins, including MMP2, MMP9, CDK4, CDK6, CyclinD1, CyclinE, RhoA, RhoC, and Cdc42. *P* < 0.05 indicates statistical significance, **P* < 0.05, ***P* < 0.01, ****P* < 0.001.

**Supplementary Figure 2. The third PLEKHH2-siRNA showed similar inhibition on cell proliferation, migration, and invasion.**

**(A)** A significant decrease in colony number was observed in cells with down-regulated PLEKHH2 compared with the controls. **(B)** The CCK-8 assay showed a time-dependent decrease in cell proliferation after PLEKHH2-siRNA transfection. The Scratch wound assay **(C)**, Transwell migration assay **(D)**, Matrigel Transwell assay **(E)**, and 3D invasion experiment **(F)** revealed that cell migration and invasion were significantly decreased in the cells transfection with PLEKHH2-siRNA. **(G)** Decreased expression of PLEKHH2 was observed in A549 and H1299 cells transfected with the third PLEKHH2-siRNA. Cells transfected with PLEKHH2-siRNA showed reduction in proliferation- and invasion-related proteins. *P* < 0.05 indicates statistical significance, **P* < 0.05, ***P* < 0.01, ****P* < 0.001.

**Supplementary Figure 3. Next-generation sequencing of mRNA results in the cells treatment with PLEKHH2-siRNA or LY294002.**

RNA-seq was performed to compare the gene expression profiles between PLEKHH2-siRNA cells and LY294002 treatment cells. **(A)** It showed the whole RNA-seq results. Gene levels of PI3K, AKT, MMP2/9, CDK4/6, CyclinD1/E, and Rho GTPase were showed in **(B)**, and there is not significantly difference between the two groups.

**Supplementary Figure 4. PLEKHH2 promotes the malignant phenotype of lung cancer cells by activating the PI3K/AKT signaling pathway.**

**(A, B)** Statistical analysis of the western blot results in Figure 4D. The analysis of A549 was shown in A, and B showed the analysis of H1299. After inhibiting the PI3K signaling pathway via LY294002, an upregulation in PLEKHH2 expression did not significantly increase p-PI3K, p-AKT, and proliferation- and invasion-related proteins. **(C, D)** Statistical analysis for the clone formation assay in Figure 4E. **(E, F)** Statistical analysis for the Matrigel Transwell assay in Figure 4G. The upregulated PLEKHH2 had no significant effect on cell proliferation and invasion ability after treatment with LY294002. *P* < 0.05 indicates statistical significance, **P* < 0.05, ***P* < 0.01, ****P* < 0.001.

**Supplementary Figure 5. PLEKHH2 activates the PI3K/AKT signaling pathway by promoting FAK phosphorylation.**

Statistical analysis of western blot in Figure 5D were shown in **(A)** and **(B)**. (**C)** and **(D)** showed the statistical analysis for the clone formation assay in Figure 5E. (**E)** and **(F)** showed the statistical analysis of Matrigel Transwell assay for Figure 5G. Following inhibition FAK phosphorylation by PF573228, the upregulation of PLEKHH2 did not increase the p-PI3K, p-AKT, or the cell proliferation and invasion ability. *P* < 0.05 indicates statistical significance, **P* < 0.05, ***P* < 0.01, ****P* < 0.001.

**Supplementary Figure 6. PLEKHH2 promotes FAK phosphorylation through its FERM domain and promotes PI3K/AKT pathway activity, and cell malignant phenotype.**

The cells transfection with PLEKHH2-ΔFERM did not significantly enhance p-FAK, p-PI3K, p-AKT, and cell malignant phenotype, compared to transfection with full-length PLEKHH2. **(A, B)** Statistical analysis for the western blot results in Figure 6E. The statistical analysis of the clone formation assay for Figure 6F were shown in (**C)** and **(D)**. **(E, F)** Statistical analysis for Matrigel Transwell assay in Figure 6H. *P* < 0.05 indicates statistical significance, **P* < 0.05, ***P* < 0.01, ****P* < 0.001.

**Supplementary Figure 7.** **PLEKHH2 binds β-arrestin1 through its FERM domain, competitively inhibits β-arrestin1 interaction with FAK, and promotes FAK phosphorylation.**

Input group was used as the internal control for normalization in the immunoprecipitation. **(A-D)** Statistical analysis for the immunoprecipitation in Figure 6D. In the A549 cell line, transfection with the full-length PLEKKH2 decreased the interaction of β-arrestin1 and FAK **(A)**. Transfection with a PLEKKH2 mutant has no effect on the binding of β-arrestin1 to FAK **(B)**. Similar results are seen in H1299 cell line **(C, D)**. **(E-H)** Statistical analysis for the immunoprecipitation in Figure 7C-7F. **(E)** PLEKKH2 or **(F)** FERM domain was transfected into A549 cells, both exhibited gradually decrease in binding with β-arrestin1 and FAK. In H1299 cell line **(G, H)**, both PLEKKH2 and its FERM had the similar effect on the binding of β-arrestin1 with FAK. *P* < 0.05 indicates statistical significance, **P* < 0.05, ***P* < 0.01, ****P* < 0.001.

**Supplementary Figure 8. The expression of PLEKHH2 in EGFR-TKI-resistant cell lines was significantly higher than that in EGFR-TKI-sensitive cell lines.**

The Gene Expression Omnibus (GEO) database was used to analyze gene expression differences between the Gefitinib-sensitive and acquired Gefitinib-resistant lung cancer cell lines. PLEKHH2 (GSE122005/GPL570/227148) is one of differential gene expression profiles.
