# Supplementary figures and images for: PLEKHH2 binds β-arrestin1 through its FERM domain, activates FAK/PI3K/AKT phosphorylation, and promotes the malignant phenotype of non-small cell lung cancer
Source: Cell Death Dis. 2022 Oct 8;13(10):858. doi: 10.1038/s41419-022-05307-5 (PMC9547923; doi:10.1038/s41419-022-05307-5)

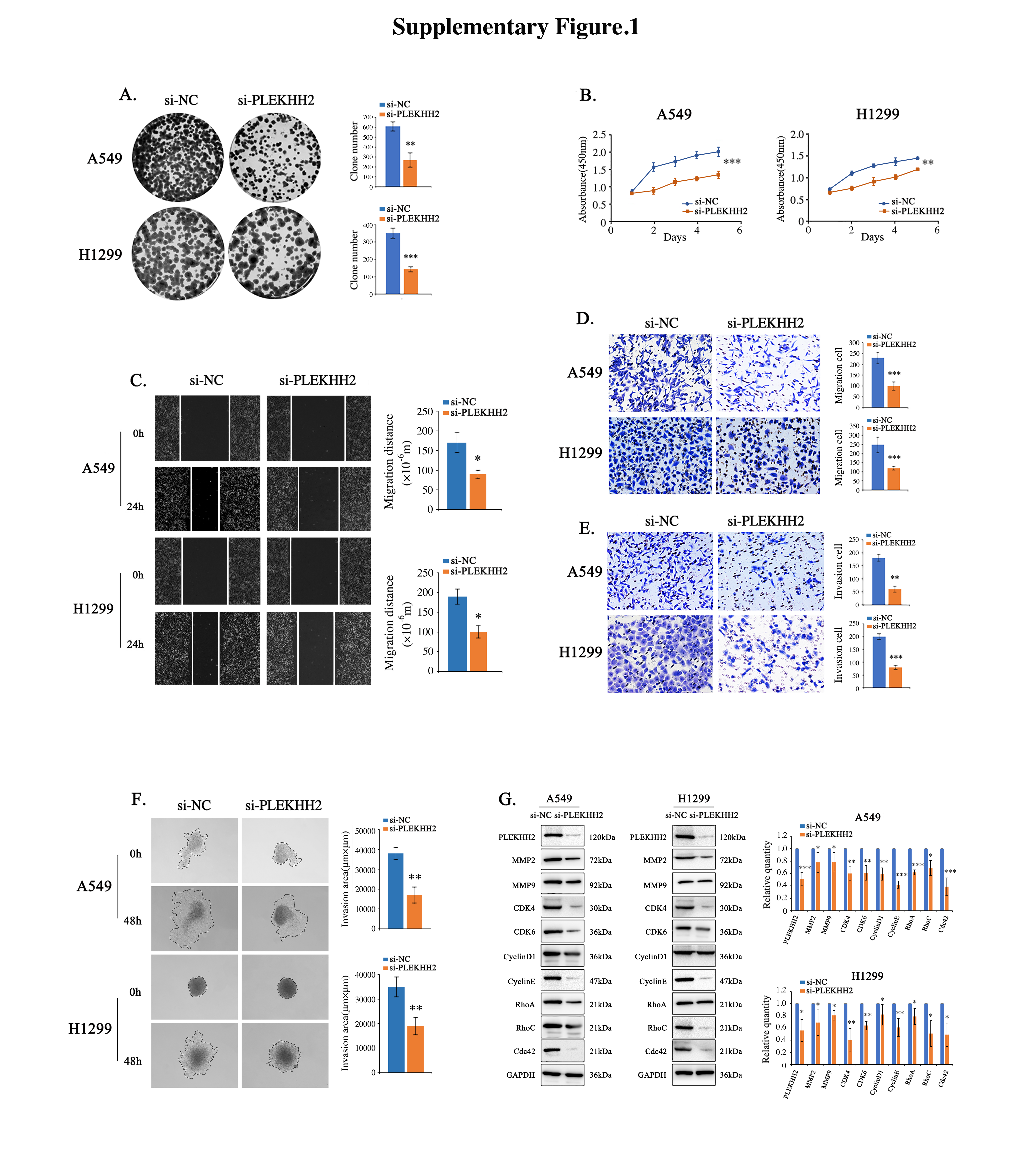

Supplement: Supplementary file 4 — Supplementary Figure 1, [file 41419_2022_5307_MOESM4_ESM.tif]

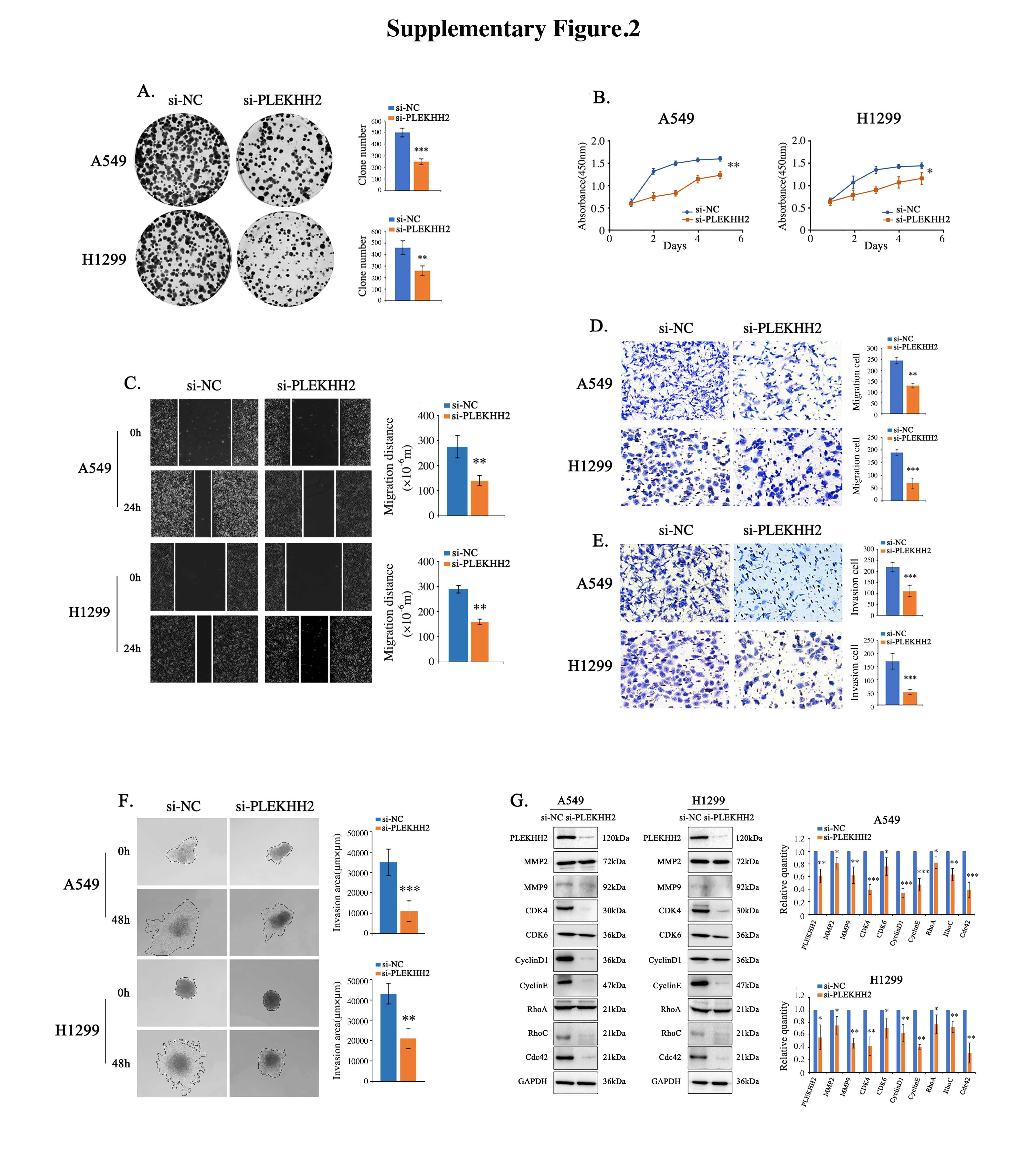

Supplement: Supplementary file 5 — Supplementary Figure 2, [file 41419_2022_5307_MOESM5_ESM.tif]

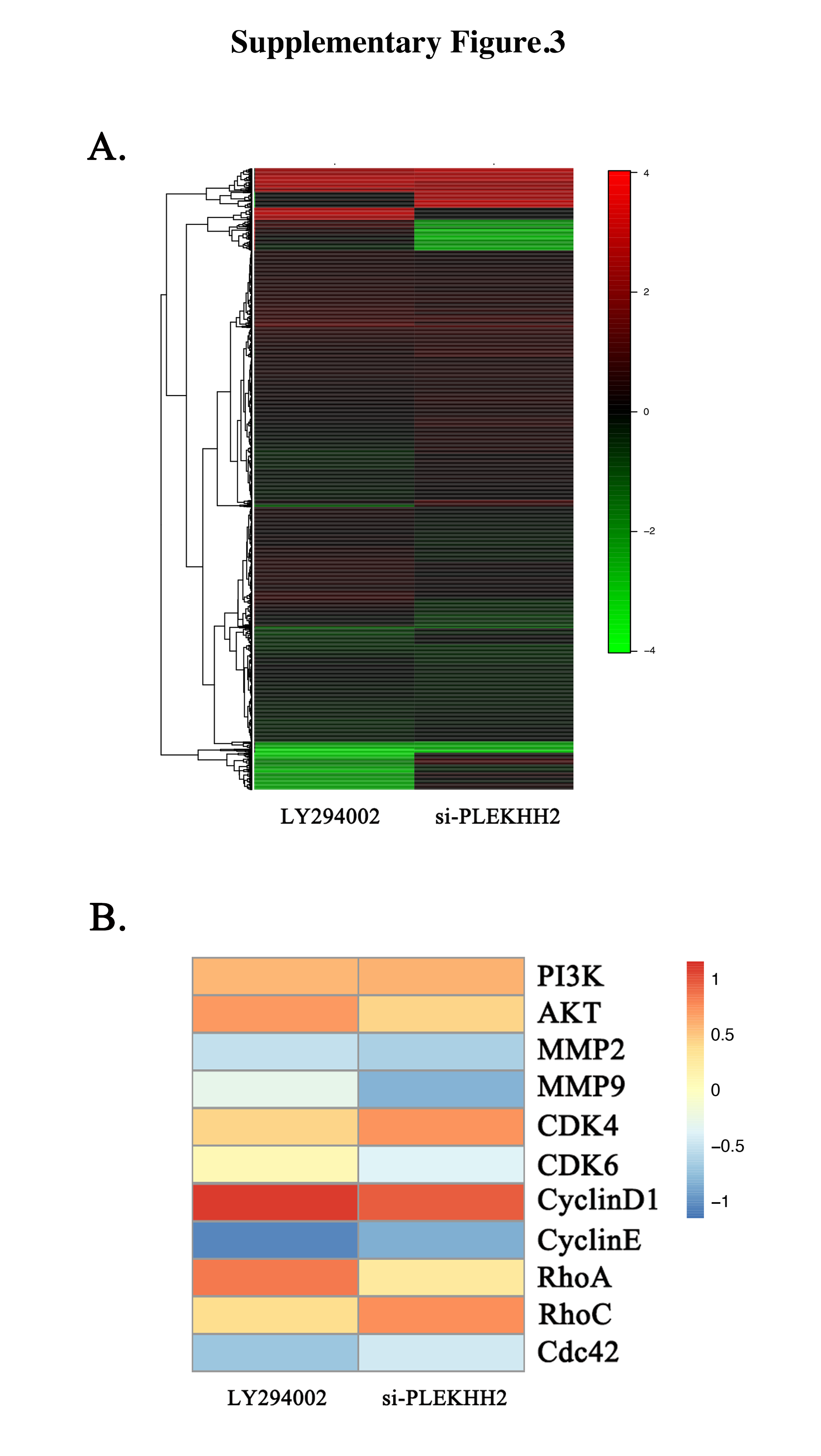

Supplement: Supplementary file 6 — Supplementary Figure 3, [file 41419_2022_5307_MOESM6_ESM.tif]

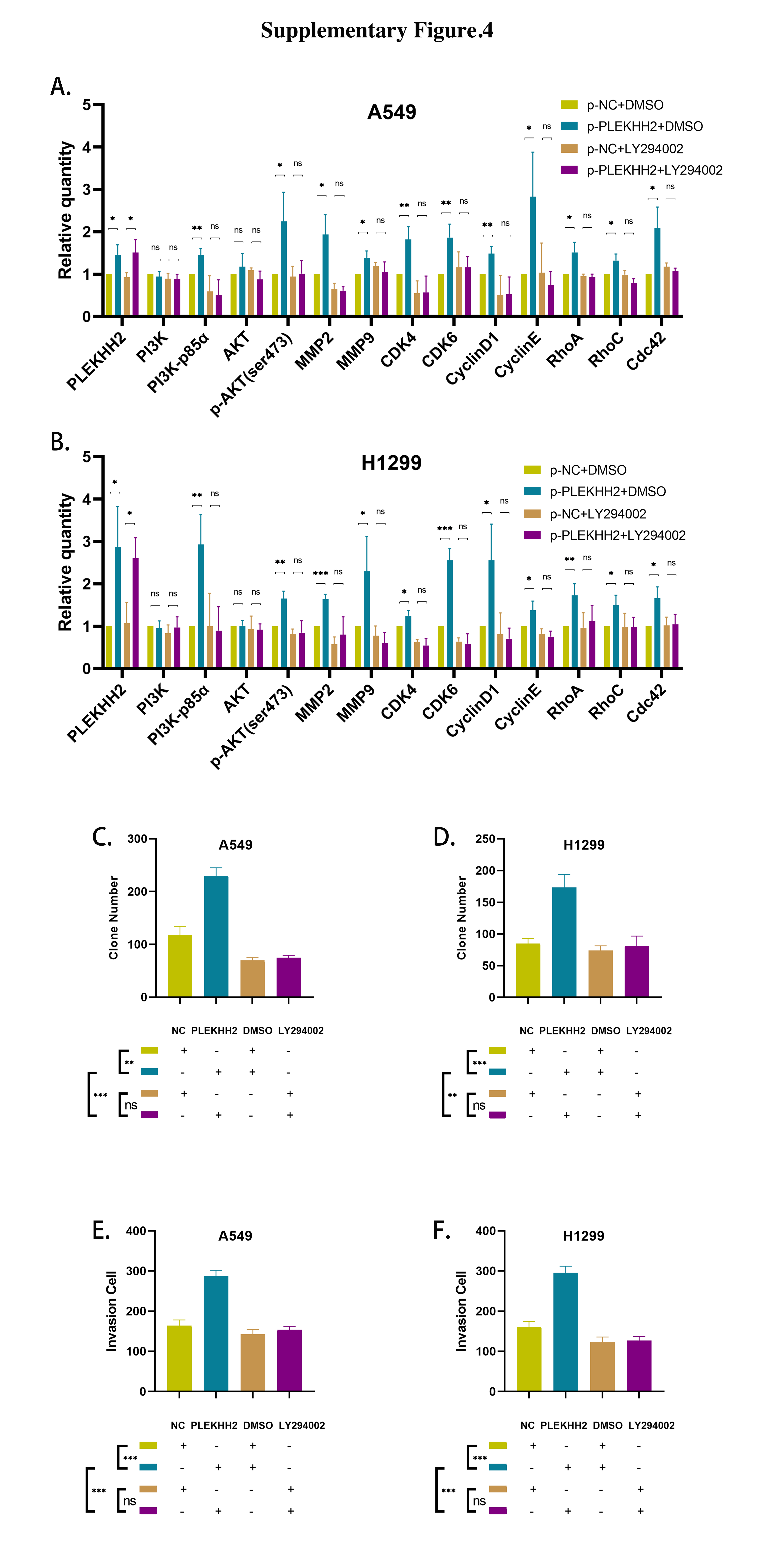

Supplement: Supplementary file 7 — Supplementary Figure 4, [file 41419_2022_5307_MOESM7_ESM.tif]

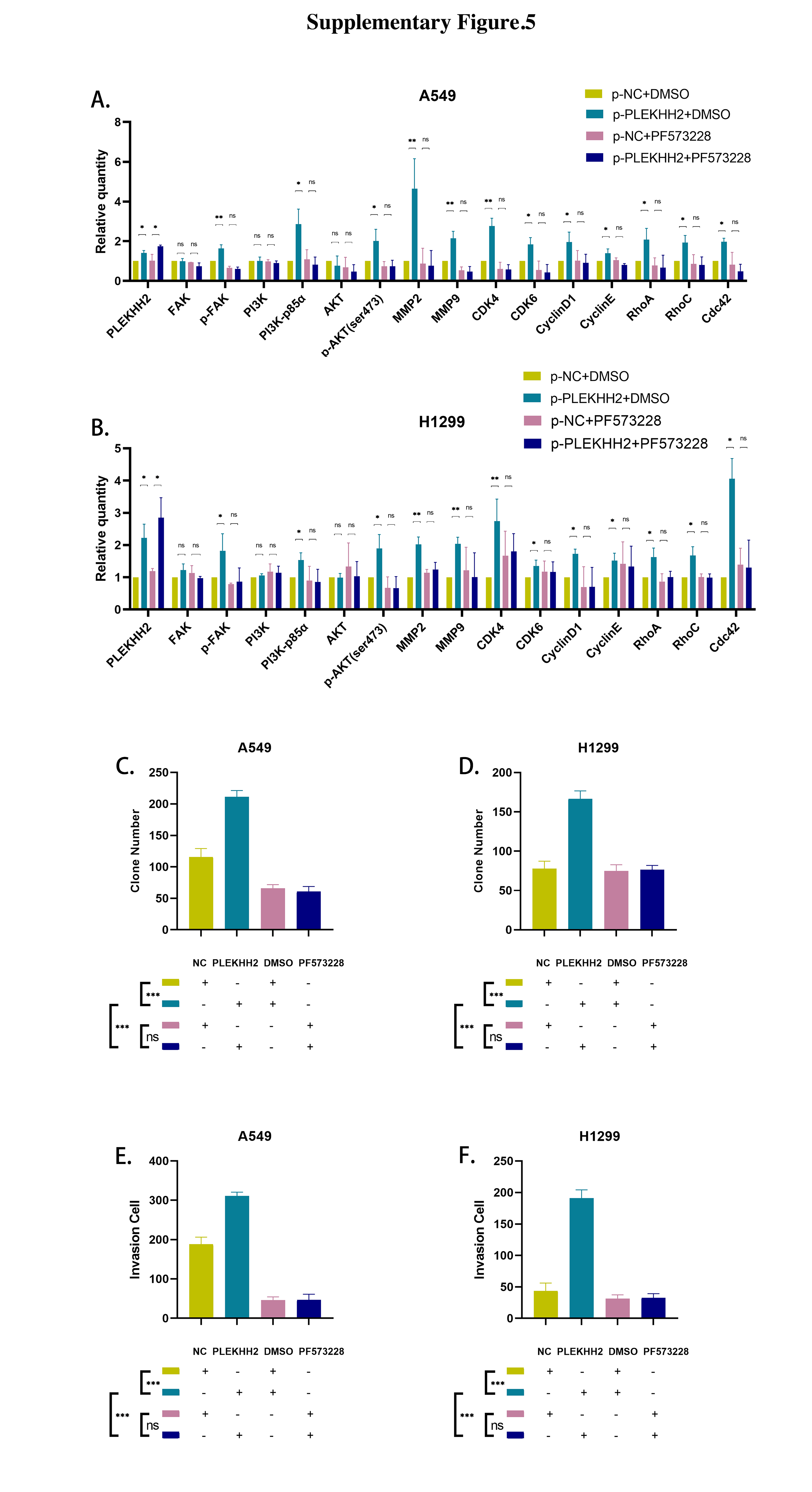

Supplement: Supplementary file 8 — Supplementary Figure 5, [file 41419_2022_5307_MOESM8_ESM.tif]

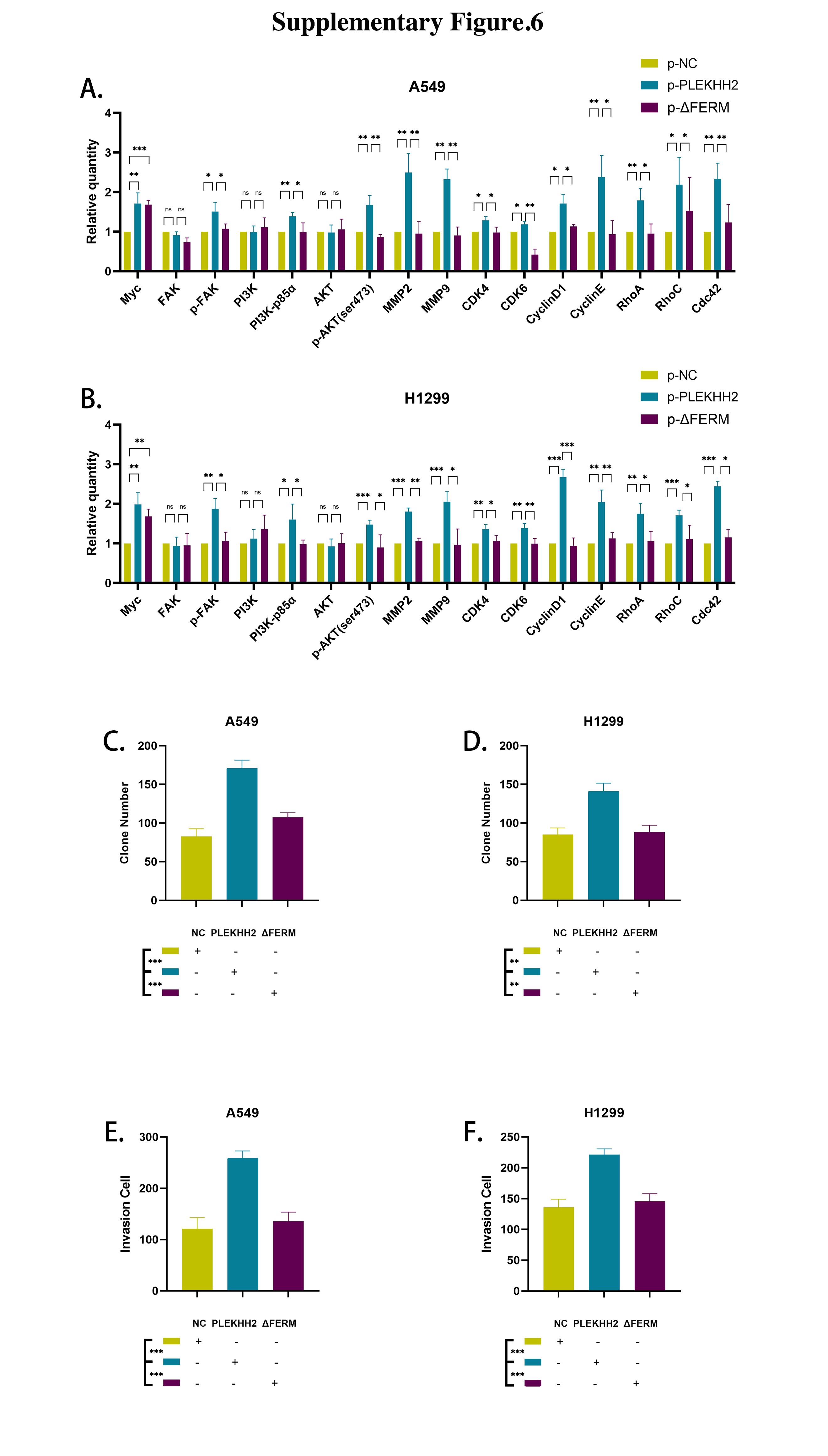

Supplement: Supplementary file 9 — Supplementary Figure 6, [file 41419_2022_5307_MOESM9_ESM.tif]

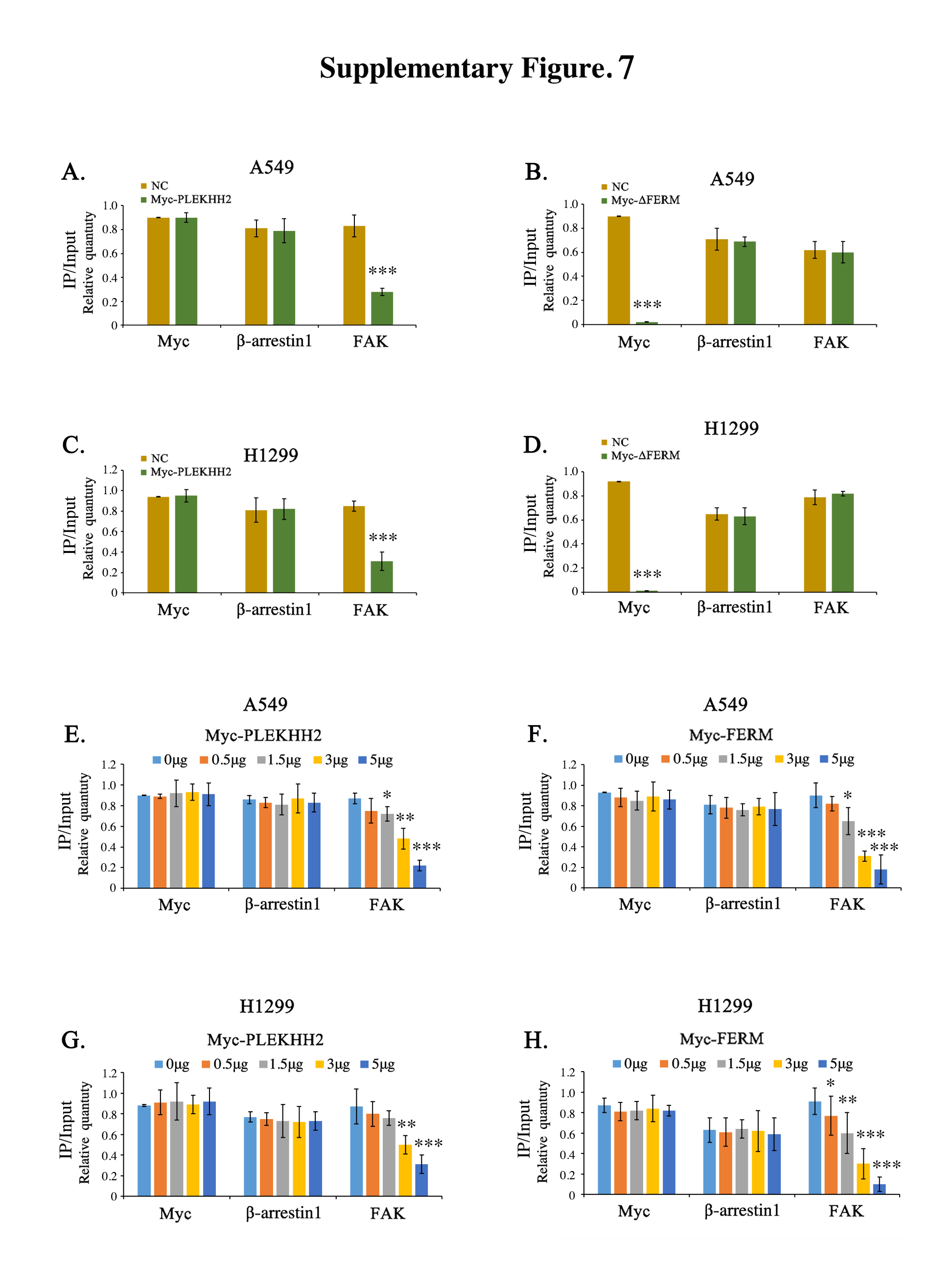

Supplement: Supplementary file 10 — Supplementary Figure 7, [file 41419_2022_5307_MOESM10_ESM.tif]

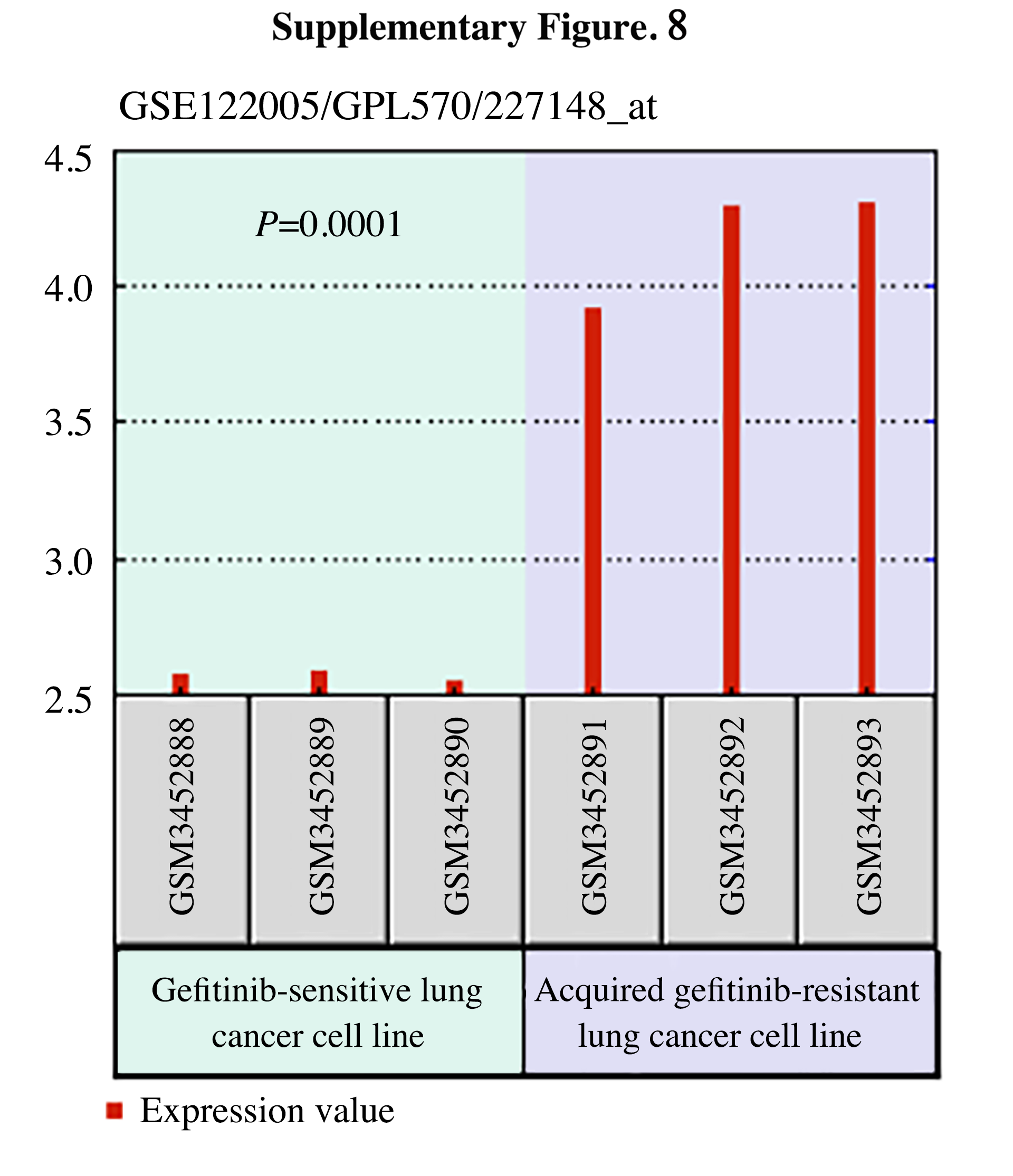

Supplement: Supplementary file 11 — Supplementary Figure 8, [file 41419_2022_5307_MOESM11_ESM.png]

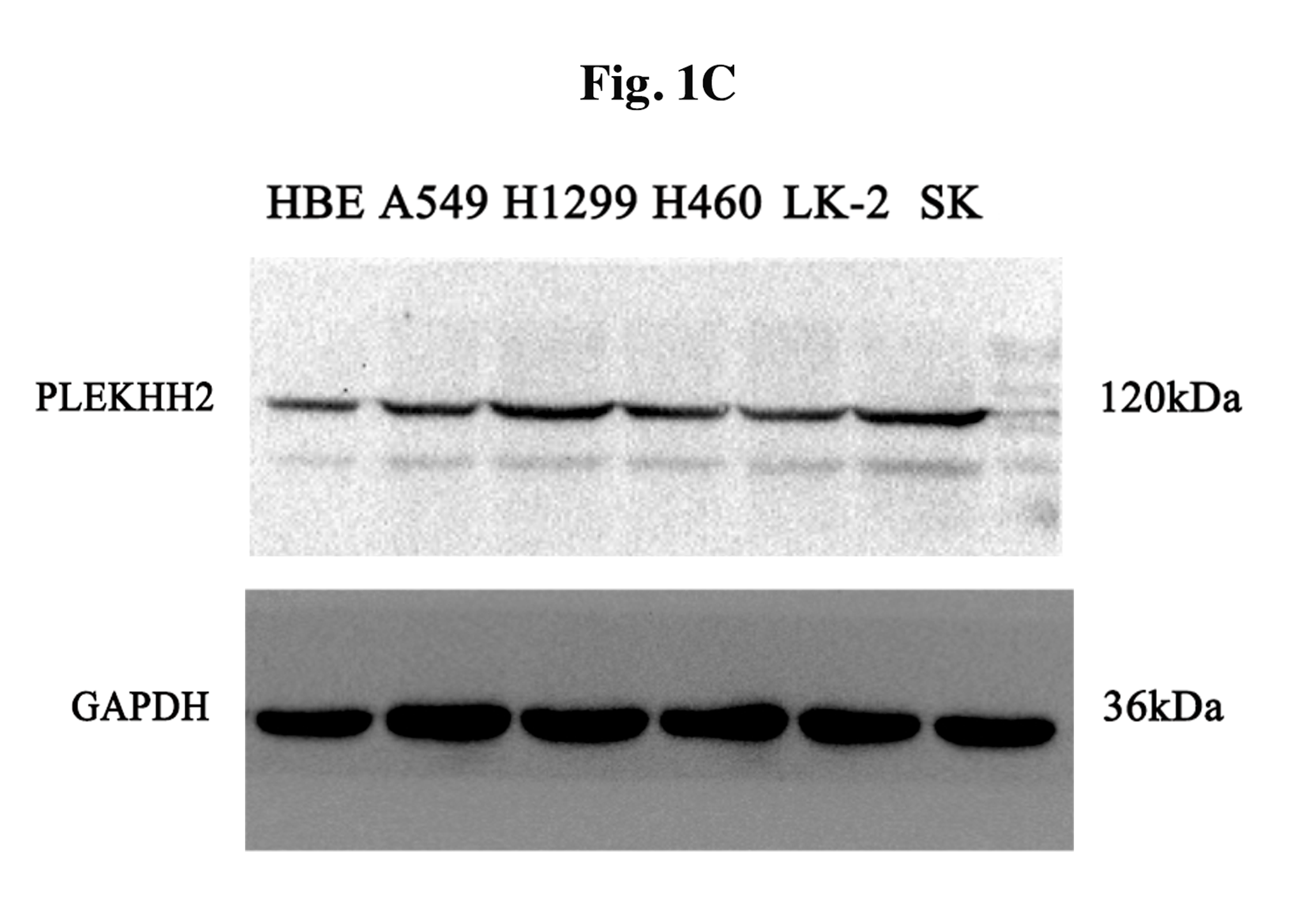

Supplement: Supplementary file 12 — Uncropped western blot-Fig1C [file 41419_2022_5307_MOESM12_ESM.tif]

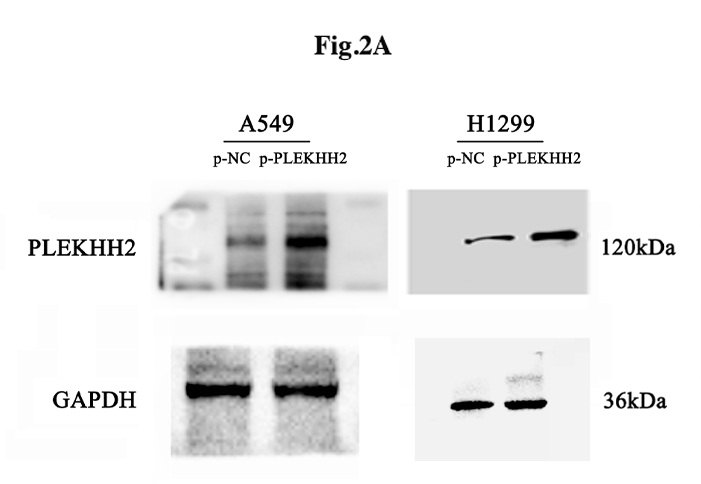

Supplement: Supplementary file 13 — Uncropped western blot-Fig2A [file 41419_2022_5307_MOESM13_ESM.tif]

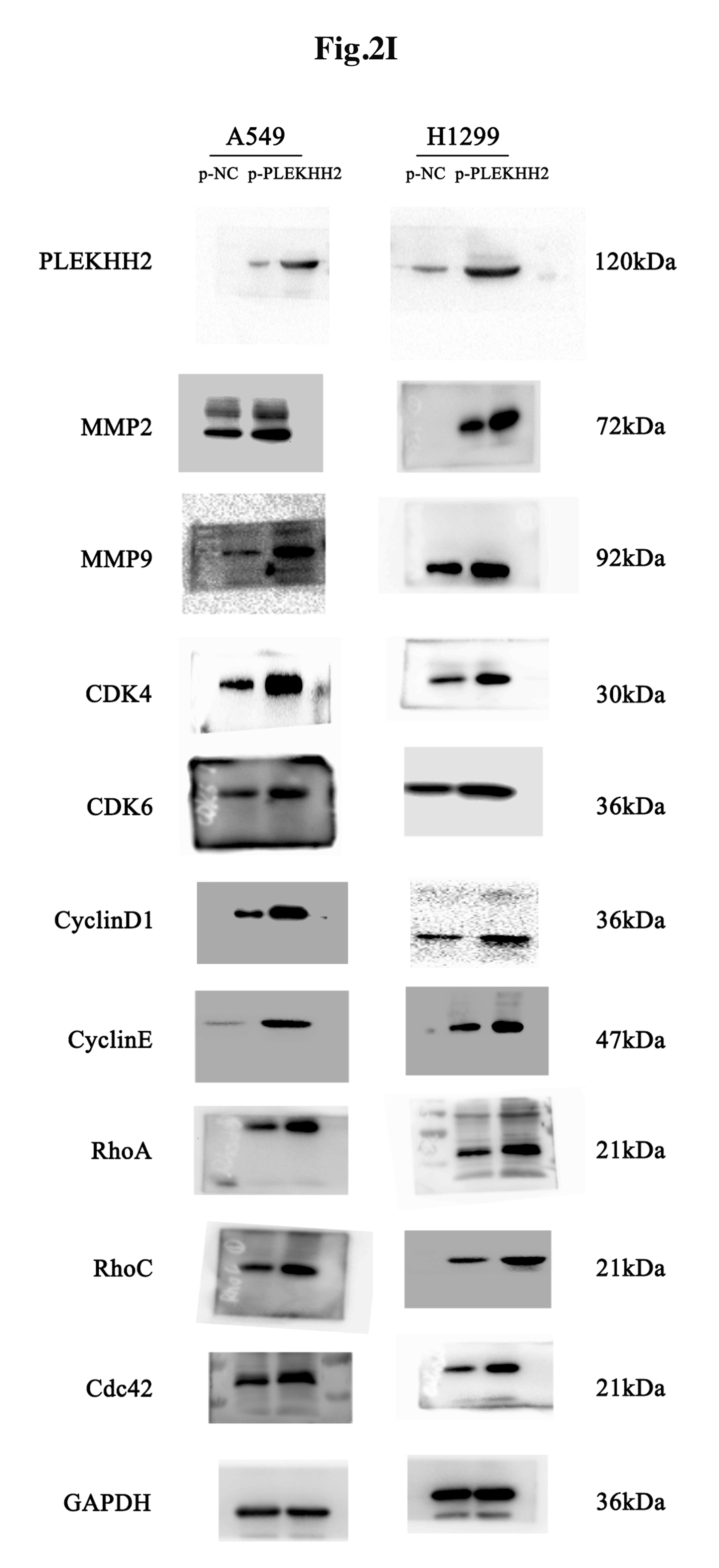

Supplement: Supplementary file 14 — Uncropped western blot-Fig2I [file 41419_2022_5307_MOESM14_ESM.tif]

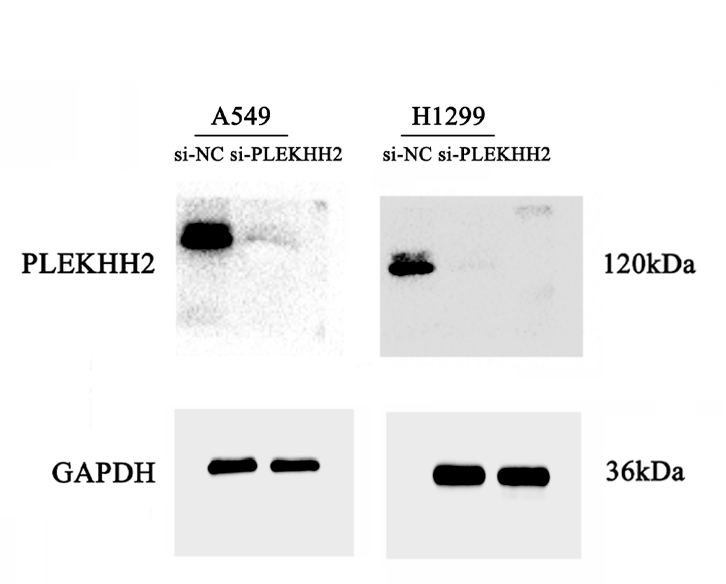

Supplement: Supplementary file 15 — Uncropped western blot-Fig3A [file 41419_2022_5307_MOESM15_ESM.tif]

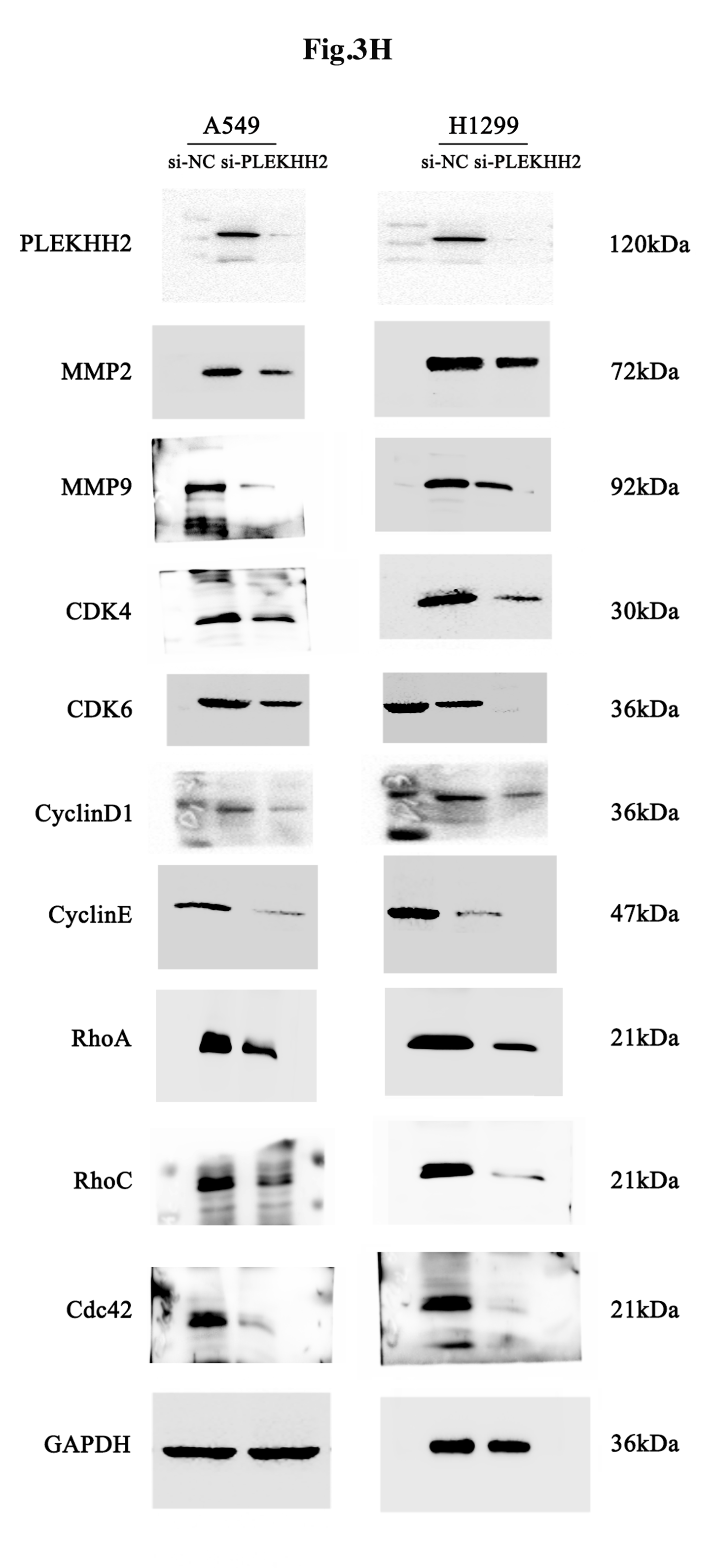

Supplement: Supplementary file 16 — Uncropped western blot-Fig3H [file 41419_2022_5307_MOESM16_ESM.tif]

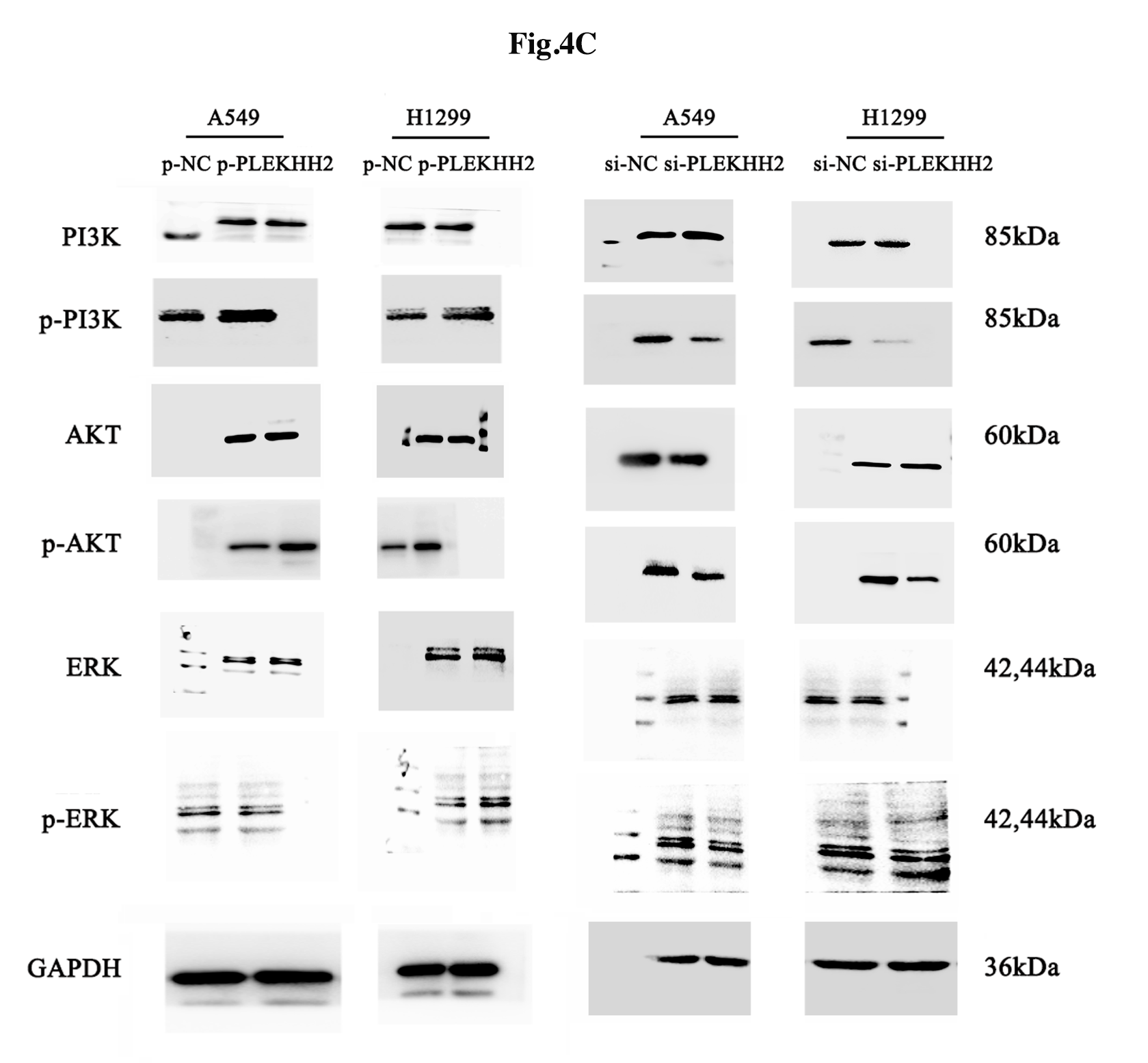

Supplement: Supplementary file 17 — Uncropped western blot-Fig4C [file 41419_2022_5307_MOESM17_ESM.tif]

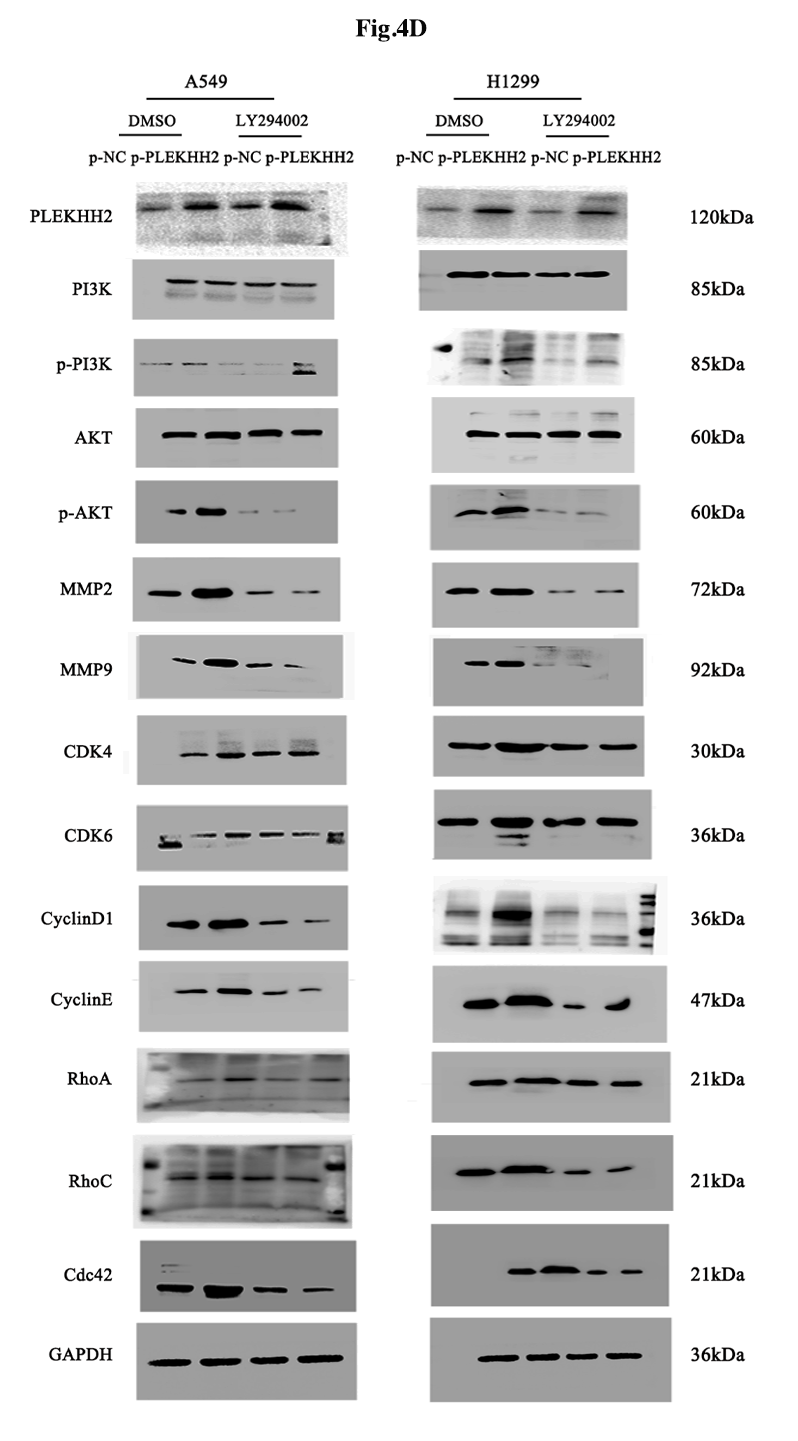

Supplement: Supplementary file 18 — Uncropped western blot-Fig4D [file 41419_2022_5307_MOESM18_ESM.tif]

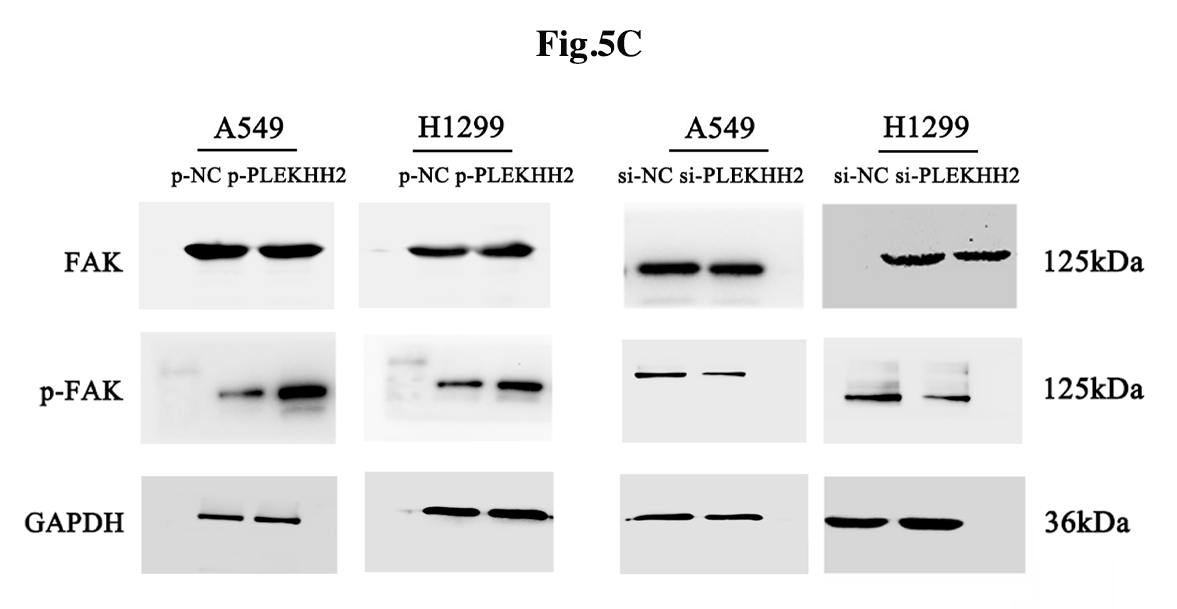

Supplement: Supplementary file 19 — Uncropped western blot-Fig5C [file 41419_2022_5307_MOESM19_ESM.tif]

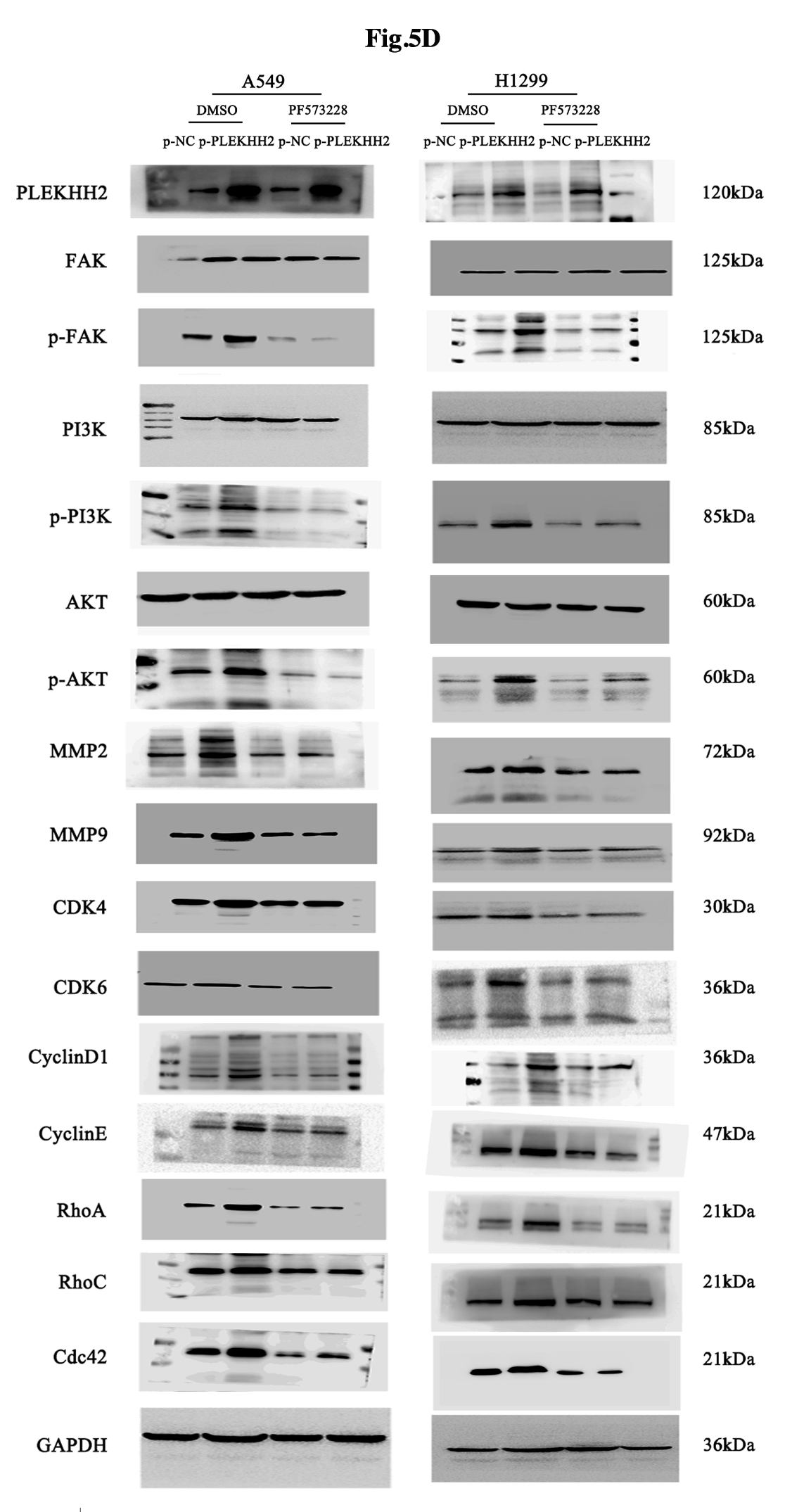

Supplement: Supplementary file 20 — Uncropped western blot-Fig5D [file 41419_2022_5307_MOESM20_ESM.tif]

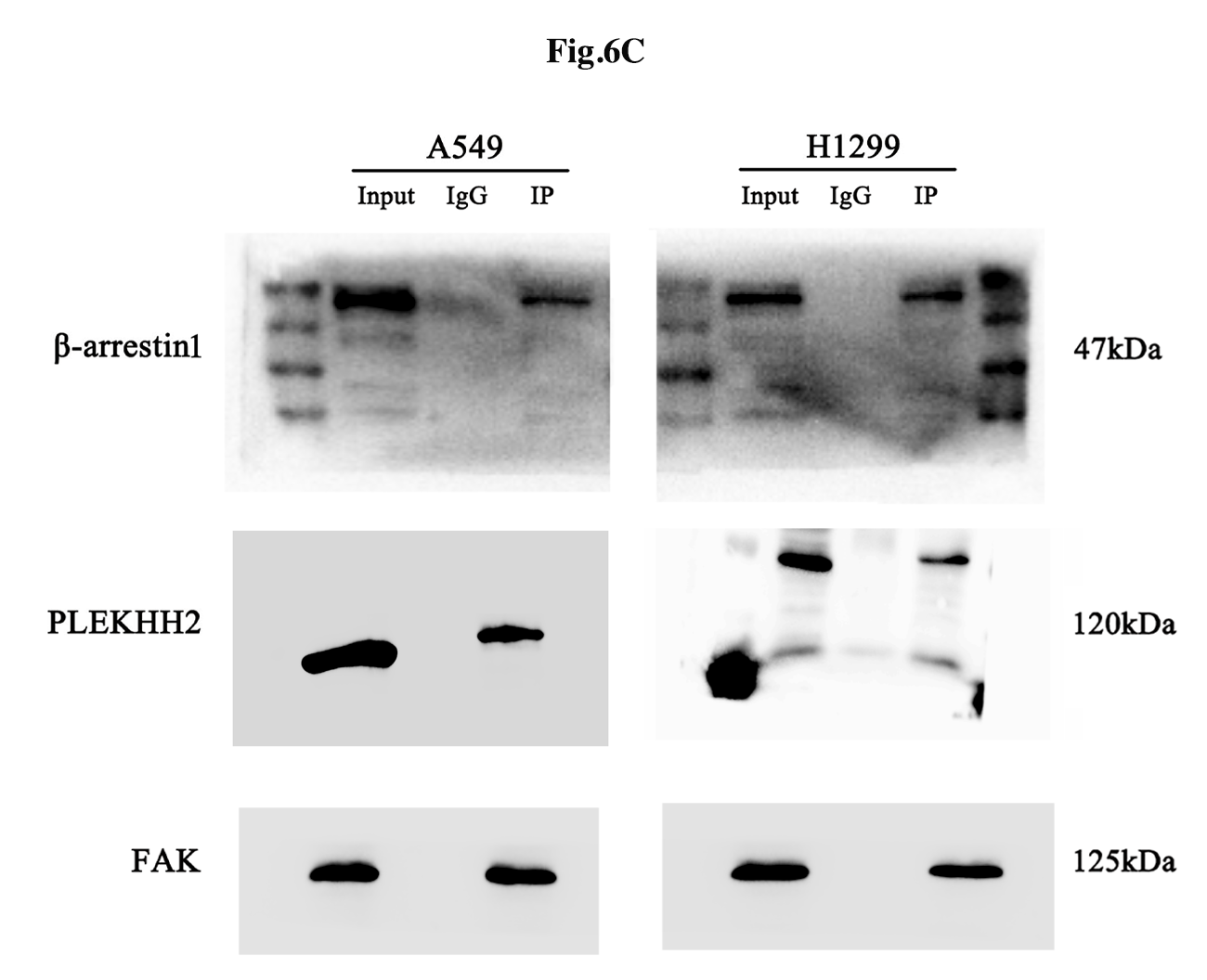

Supplement: Supplementary file 21 — Uncropped western blot-Fig6C [file 41419_2022_5307_MOESM21_ESM.tif]

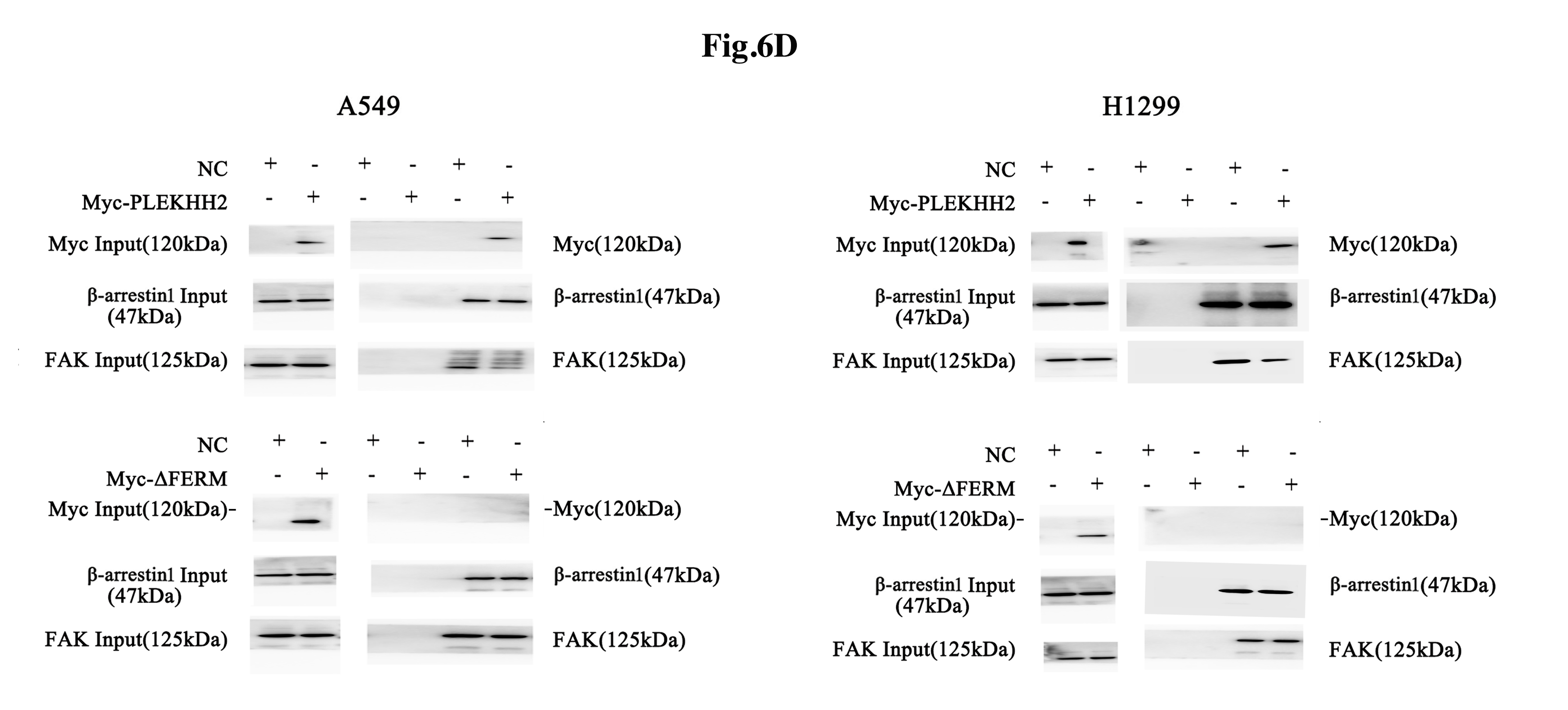

Supplement: Supplementary file 22 — Uncropped western blot-Fig6D [file 41419_2022_5307_MOESM22_ESM.tif]

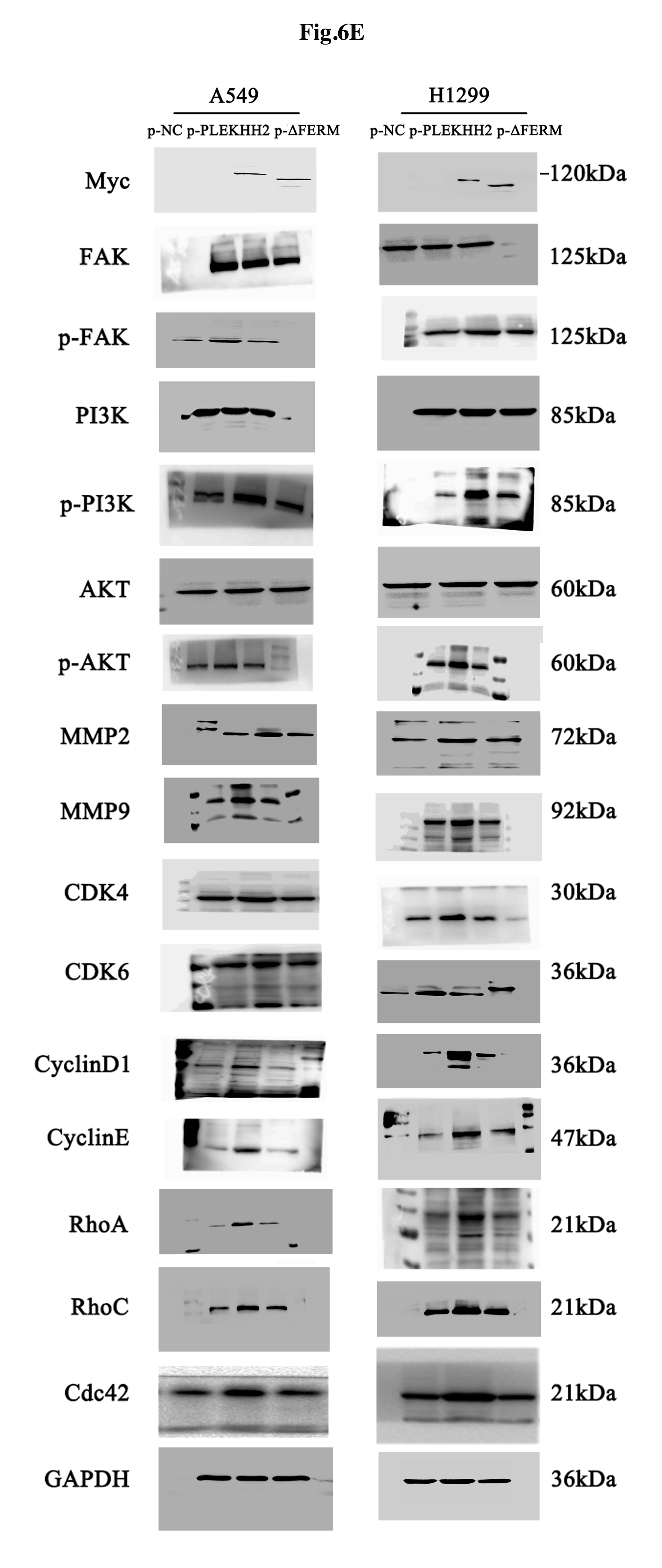

Supplement: Supplementary file 23 — Uncropped western blot-Fig6E [file 41419_2022_5307_MOESM23_ESM.tif]

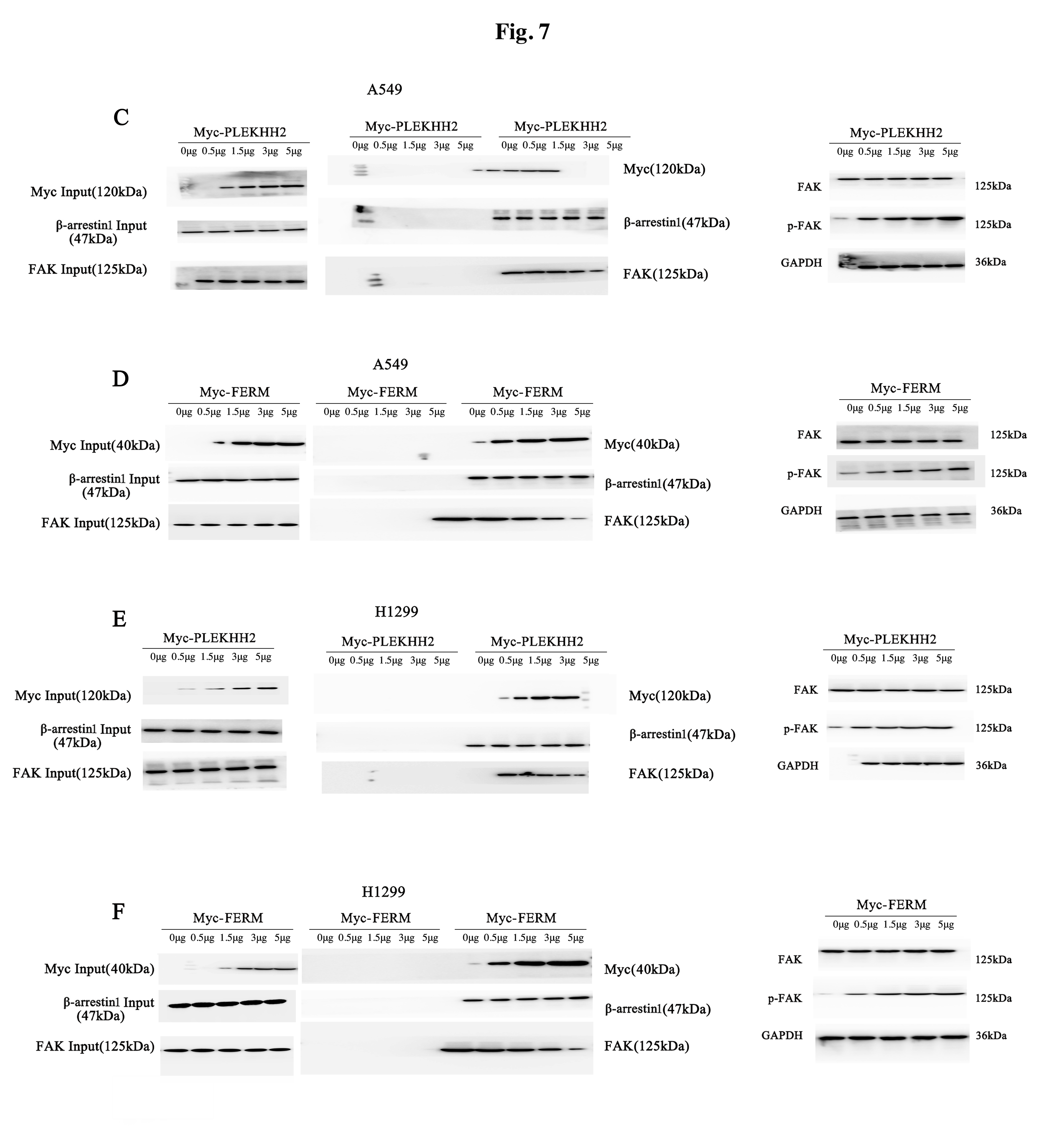

Supplement: Supplementary file 24 — Uncropped western blot-Fig7 [file 41419_2022_5307_MOESM24_ESM.tif]

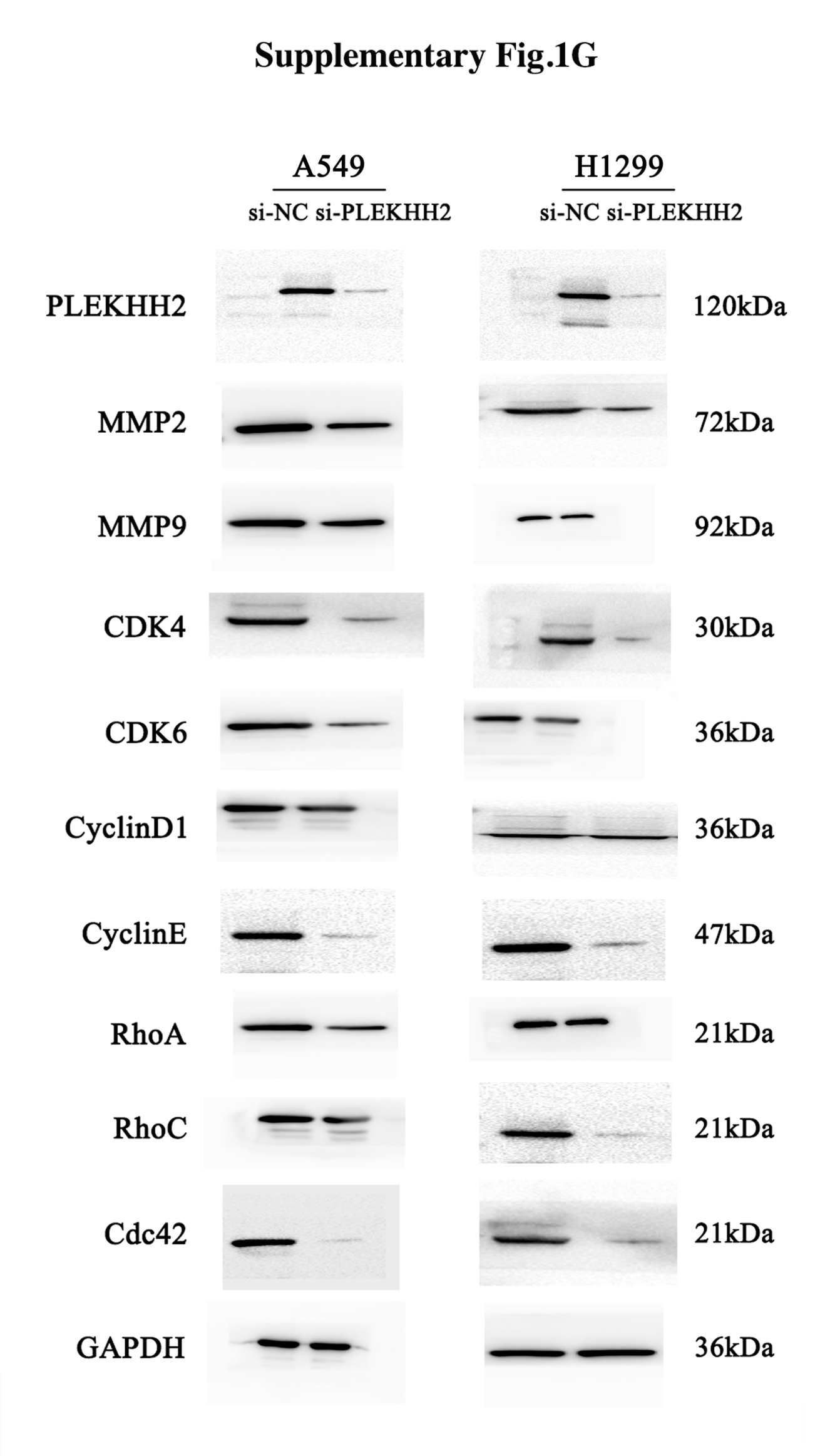

Supplement: Supplementary file 25 — Uncropped western blot-Supplementary Fig1G [file 41419_2022_5307_MOESM25_ESM.tif]

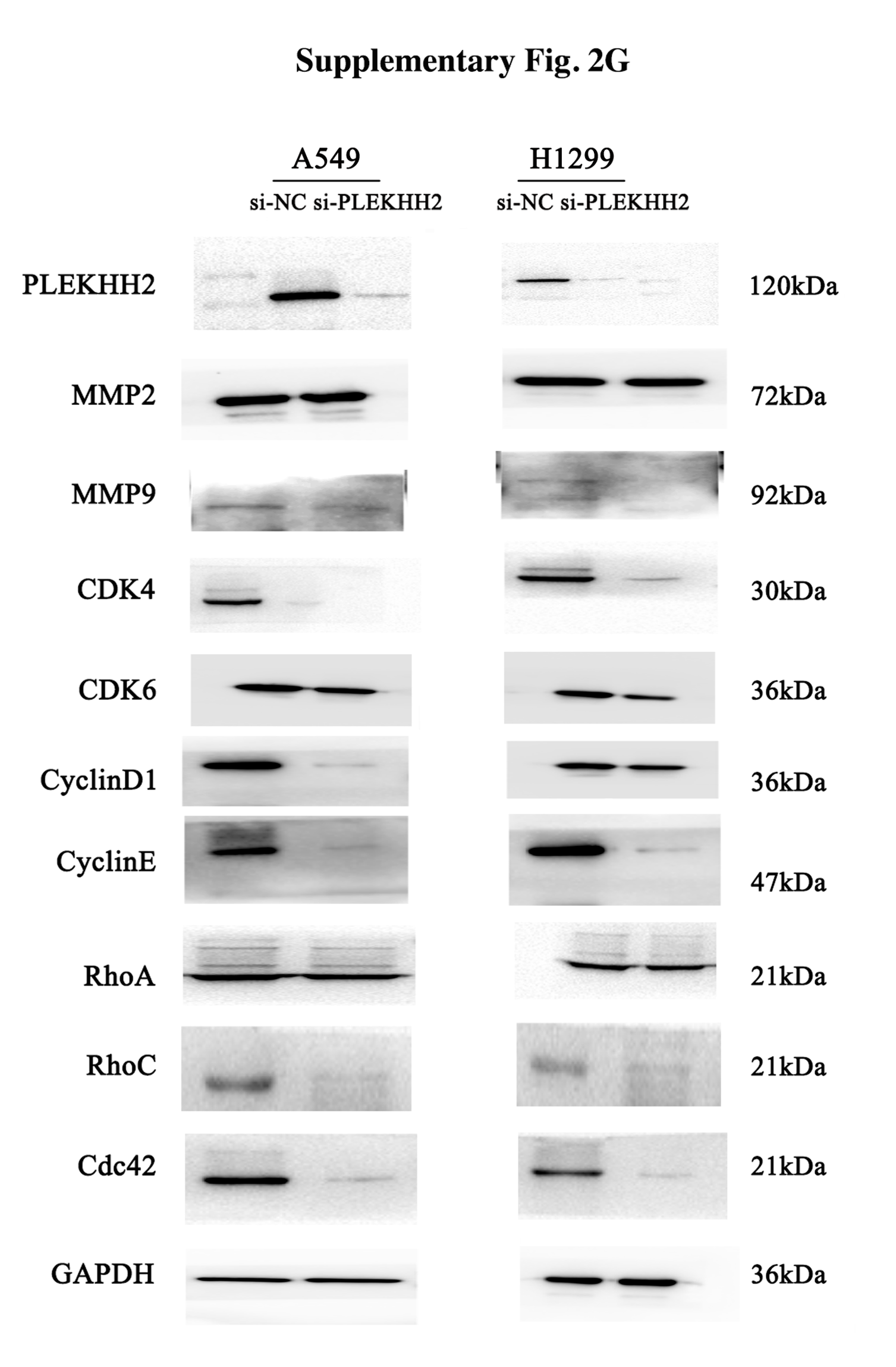

Supplement: Supplementary file 26 — Uncropped western blot-Supplementary Fig2G [file 41419_2022_5307_MOESM26_ESM.tif]
